# Association between hospital frailty risk score, risk of sepsis and adverse outcomes across all adult ages

**DOI:** 10.1371/journal.pone.0342790

**Published:** 2026-02-13

**Authors:** Huda Kutrani, Jim Briggs, David Prytherch, Claire Spice

**Affiliations:** 1 Centre for Healthcare Modelling and Informatics, University of Portsmouth, Portsmouth, United Kingdom; 2 Faculty of Public Health, University of Benghazi, Benghazi, Libya; 3 Queen Alexandra Hospital, Portsmouth Hospitals University Trust, Portsmouth, United Kingdom; Gabriele d'Annunzio University of Chieti and Pescara: Universita degli Studi Gabriele d'Annunzio Chieti Pescara, ITALY

## Abstract

**Background:**

Hospital Frailty Risk Score (HFRS) is commonly used to identify frailty risk and predict poor outcomes. Frailty and sepsis are associated with poor outcomes. This study aimed to evaluate the association between HFRS, risk of sepsis and poor health outcomes across all adult ages.

**Methods:**

A retrospective cohort study analysed data from Queen Alexandra Hospital, a major acute hospital in the UK, covering the period from January 1, 2010, to December 31, 2019. It included patients aged 16 and older. The Hospital Frailty Risk Score (HFRS) was used to identify patients at risk of frailty. The Suspicion of Sepsis (SOS) codes and the National Early Warning Score (NEWS) were used to identify patients at risk of sepsis. Logistic Regression with interaction models were developed to examine the associations between HFRS, risk of sepsis and poor outcomes (length of stay and in-hospital mortality) across all adult ages.

**Results:**

Patients with higher frailty risk and sepsis-risk-positive groups had longer length of stay and higher risk of in-hospital mortality compared to the sepsis-risk-negative groups. Interaction between intermediate or high frailty risk and sepsis-risk-positive (SOS codes present, NEWS≥7, and SOS codes present with NEWS≥7 groups) was significant for all periods of length of stay and all periods of in-hospital mortality (P < 0.001). Moreover, sepsis-risk-positive groups had a greater impact on the predictive power of HFRS to predict long LOS and in-hospital mortality compared to the sepsis-risk-negative groups.

**Conclusion:**

This study concluded that there is a strong association between risk of frailty (identified with HFRS), risk of sepsis, and poor outcomes in urgently hospitalised adults of all ages.

## Introduction

Frailty is a medical syndrome characterised by reduced physiologic function and increased dependency; it is associated with poor outcomes [1,2]. Although frailty is most common in older people and increases with age [1,3–5], it has been demonstrated across almost all age groups [1,6–9]. It is common and rising, for example, in England, frailty prevalence increased from 26.5% in 2006 to 38.9% in 2017 for adult patients aged 50 years and above [10].

Multiple tools have been developed to identify people with, or at a high risk of frailty, but many require direct assessment, such as the Frailty index [11] and functional judgement scales such as the Clinical Frailty Scale [12]. This can be challenging to assess in all patients admitted to hospital [13–16]. The Hospital Frailty Risk Score (HFRS) is a frailty risk tool based on ICD-10 codes related to frailty [17], not requiring direct assessment by a clinician, and for medical inpatients is known to perform at least as well as or better than many other tools to measure frailty [17,18].

Sepsis is defined by international consensus as a “life-threatening organ dysfunction caused by a dysregulated host response to infection” [19], is a major healthcare concern worldwide, reducing quality of life and causing in-hospital mortality [19–21]. People with sepsis are at high risk of illness or death; in the UK (2014), sepsis caused about 44,000 deaths per year and there were an estimated 150,000 cases [22]. Sepsis is increasing, in part due to ageing populations with increasing morbidities and frailty [19].

Frailty is associated with both an increased risk of developing sepsis and poorer outcomes for those who have both sepsis and frailty [23–27]. Both frailty and sepsis increase healthcare costs and resource use and reduce quality of life [23,25,26].

Gulliford et al. (2020) evaluated an association of age, gender and frailty as risk factors for the probability of sepsis in primary care. They assessed frailty with the Electronic Frailty Index which uses Primary Care codes and found that, at all ages, frailty was associated with a greater risk of sepsis, which rose with increasing severity of frailty [24]. In addition, Mahalingam et al. (2019) studied the associations between frailty and long-term risk of sepsis in a large group of adults in the community and demonstrated that frailty was associated with the risk of first-sepsis hospitalizations and sepsis 30-day mortality [25]. For hospital-based populations, there have been few studies using HFRS and none in a general hospital population. Sarría-Santamera et al. (2022) found that older surgical patients with intermediate or high frailty risk had a higher risk of sepsis compared to the low frailty risk group [23]. Patients with high frailty scores in a group of all ages discharged with chronic pancreatitis had a higher risk of sepsis [27].

Early detection and treatment of frailty and sepsis can assist in reducing mortality and illness [26], and reduce hospital resource use [23,25,26]. Routinely applicable risk of frailty scores, without need for clinical assessment, may be of benefit both in clinical and research settings. This study aimed to evaluate the association between risk of frailty (identified with HFRS), risk of sepsis and the outcomes of in-hospital mortality and length of stay in a large group of urgently hospitalised (non-elective) adults of all ages.

## Materials and methods

### Study design and participants

This study follows similar methods to those of our team’s previous work [28–30], but we summarise them here for convenience. This retrospective cohort study included non-elective patients aged 16 years and older admitted from 1st January 2010–31^st^ December 2019 to Queen Alexandra Hospital in Portsmouth, UK (575,045 admissions).

**Fig 1:**
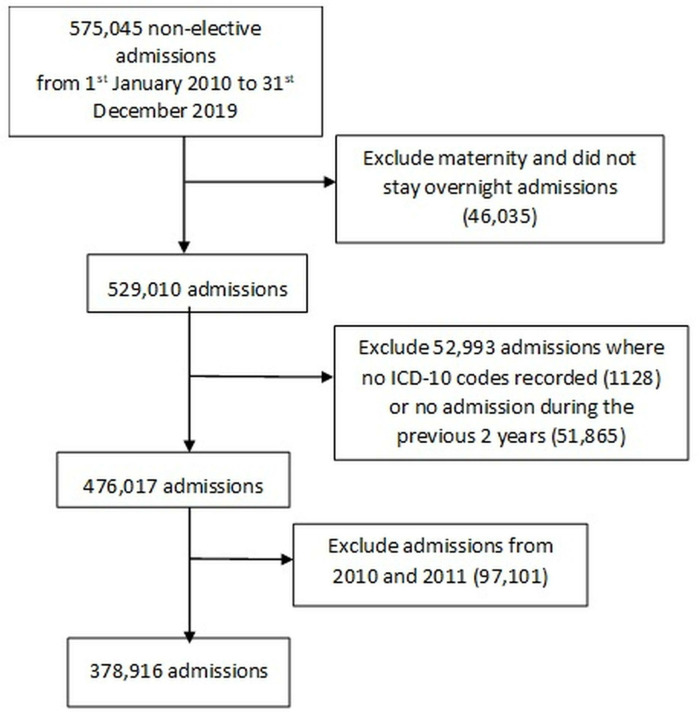
Flowchart showing the inclusion and exclusion criteria for the study.

Since the calculation of HFRS relies on having ICD-10 codes from 2 previous years’ data for optimal construction of the HFRS [31], analysis was limited to patients admitted from 1^st^ January 2012–31^st^ December 2019. Patients with no hospital admissions in the preceding two years were excluded as per the validated methodology for HFRS construction, as insufficient ICD-10 information was available to calculate HFRS for this group. It should be noted that this necessitated the exclusion of 51,865 patients without previous hospitalisation, who may represent a healthier cohort with lower frailty risk, and thus could not be assigned an HFRS. We excluded maternity cases, and we also excluded certain types of admission from the study as described in Fig 1.

### Identifying risk of frailty

We used the Hospital Frailty Risk Score (HFRS) to evaluate risk of frailty levels. The HFRS is a frailty assessment tool, calculated based on 109 International Classification of Diseases 10th revision (ICD-10) diagnosis codes related to frailty. It was developed and validated in secondary care within the NHS for older people to identify frailty risk and predict poor outcomes [17]. Hospital Frailty Risk Scores were categorised into low frailty risk (less than 5), intermediate frailty risk (5–15), and high frailty risk (>15) as per the original study.

We calculated the “modified” HFRS (by excluding the current “index” admission from the HFRS calculation) for each patient using the methodology described by the original study [17], with optimal construction of the HFRS by adding the weighted points together for each code present from any diagnosis recorded from previous admissions during the previous two years, per Street et al [31]. We used the “modified” HFRS to identify patients at risk of frailty at the time of hospital admission. This replicates the information that would be available at the time of a patient’s admission, before any diagnoses have been coded, and thus allows us to evaluate HFRS in the way it could be used operationally.

We also conducted an analysis based on the original HFRS (by including the current “index” admission into the HFRS calculation) to determine whether using a modified version, which is more applicable at the point of admission, altered the findings.

### Identifying risk of sepsis

We used the Suspicion of Sepsis (SOS) coding set and the National Early Warning Score (NEWS) to assign patients’ probabilities of sepsis. The Suspicion of Sepsis (SOS) coding set is a list of diagnostic codes (ICD) related to sepsis and septic shock drawn from the International Classification of Diseases, Ninth Revision, Clinical Modification (ICD-9-CM) and the International Classification of Diseases, Tenth Revision (ICD-10) codes (codes known to be infective bacterial pathogens that can cause sepsis) [19,21,32,33]. According to the presence/absence of suspicion of sepsis (SOS) codes, we can identify a patient’s infection status [24,32,33]. One or more of the SOS codes present in the medical record indicate a patient is at risk of sepsis. Therefore, the presence of SOS codes was classified “sepsis-risk-positive”, and the absence of any SOS codes as “sepsis-risk-negative”.

The Royal College of Physicians (2017) reported that “the National Early Warning Score (NEWS) has also been endorsed as the recommended early warning system to detect acute clinical illness/deterioration due to sepsis in patients with an infection or at risk of infection” [34]. According to the National Early Warning Score (NEWS)2 report “NEWS score≥5 in patients with a known infection, signs or symptoms of infection, or at high risk of infection, is most likely to represent sepsis” and score≥7 indicates patients more likely have sepsis [34]. Other studies also suggested that NEWS2 was effective at identifying patients at risk of sepsis [35–38]. According to these, we selected two NEWS thresholds of 5 or more (NEWS≥5) and 7 or more (NEWS≥7) to identify patients at risk of sepsis. Whilst the optimal approach for identifying sepsis would combine an elevated NEWS2 score with clinical evidence of suspected or confirmed-infection the approach we used was necessary due to the challenges of retrospectively extracting reliable infection data from electronic health records. Therefore, any admission with a NEWS score≥5 or 7 was classified as “sepsis-risk-positive”, and NEWS<5 or 7 as “sepsis-risk-negative”, for each threshold, acknowledging that not all elevated NEWS2 scores necessarily indicate sepsis and some may reflect other acute physiological deterioration. We calculated NEWS from vital signs for each patient using the methodology described by the Royal College of Physicians [34]. Vital signs studied were the first routine data gathered from a patient after admission.

For our analysis, we defined 7 groups of patients, as follows:

Group A: SOS codes were present (n = 132,786 admissions).
Group B: NEWS score≥7 (13,007 admissions).
Group C: SOS codes were present AND NEWS score≥7 (9,572 admissions).
Group D: no SOS codes were present AND NEWS score<7 (170,216 admissions).
Group E: NEWS score≥5 (34,978 admissions).
Group F: SOS codes were present AND NEWS score≥5 (23,787 admissions).
Group G: no SOS codes were present AND NEWS score<5 (162,460 admissions).

Groups A, B, C, E, and F are considered sepsis-risk-positive, whereas groups D and G are considered sepsis-risk-negative.

### Study outcomes

The outcomes considered in this study were length of stay (LOS) and in-hospital mortality (death at discharge). We used nine periods of LOS and eight periods of in-hospital mortality, as shown in Table 1, to identify any nuances that average length of stay or total in-hospital mortality would mask.

**Table 1:** Prediction periods of length of stay and in-hospital mortality.

9 periods of LOS	8 periods of in-hospital mortality
LOS > 3 days	3-day mortality
LOS > 7 days	7-day mortality
LOS > 10 days	10-day mortality
LOS > 14 days	14-day mortality
LOS > 21 days	30-day mortality
LOS > 30 days	60-day mortality
LOS > 45 days	90-day mortality
LOS > 60 days	6-month mortality
LOS > 90 days	

### Statistical analysis

We summarised patients’ characteristics using mean and standard deviation, median and interquartile range (IQR) for continuous variables, and frequency and percentage for categorical variables. We compared patients’ characteristics and outcomes between the sepsis-risk-positive and the sepsis-risk-negative groups using a chi-square test for categorical characteristics and an independent sample t test or Mann–Whitney U test for continuous variables. P values <0.05 were considered statistically significant.

Using logistic regression with interaction, models were developed for each of the nine LOSs and eight in-hospital mortality periods for each of the seven patient groups. The interaction between Hospital Frailty Risk Score categories (low, intermediate, high) and sepsis-risk groups (sepsis-risk-positive or sepsis-risk-negative) was assessed to determine whether the association and interaction between frailty and longer LOS or higher in-hospital mortality differed according to sepsis-risk groups. The reference categories for the interaction terms were low frailty risk (HFRS<5) and sepsis-risk-negative (SOS codes-absent or NEWS<5 or 7).

Associations between HFRS categories and the sepsis-risk-positive status across each length of stay period, as well as in-hospital mortality, were evaluated using odds ratios (ORs) with 95% confidence intervals (CIs). Model performance and discrimination were assessed using c-statistics with 95% confidence intervals.

Predicted Probability Analysis: To further interpret the interaction effects, we calculated the model-predicted probabilities of LOS and in-hospital mortality for combinations of HFRS categories and two key clinical indicators: Suspicion of Sepsis (SOS) codes and National Early Warning Score (NEWS). Predicted probabilities were estimated from the logistic regression models. It is a value between 0 and 1 that indicates the effect of SOS codes-present or NEWS≥5 or 7 on the HFRS categories to predict longer LOS and in-hospital mortality. This approach allowed us to quantify and visualize how the presence of sepsis-risk-positive (either SOS codes were present or NEWS≥5 or 7) affects risk of frailty (HFRS) in predicting LOS or in-hospital mortality.

We used Restricted Cubic Splines (RCS) to test the non-linear relationship between continuous modified HFRS and all periods of LOS and in-hospital mortality. Dose-response curves were used to visualize the relationship. Data manipulation and logistic regression modelling were performed using RStudio version 4.2.1.

### Ethics

The study included patients aged 16 years and older who had not registered for the national data opt-out for their data being used for research. Only data from individuals who had not opted out of data sharing were included. We accessed data from the Portsmouth CORE-D routine care data repository on 1st November 2022. All data were fully anonymized before being made available to the research team, and no information that could identify individual patients was available to researchers during or after data collection.

The dataset used in this study was covered by existing ethical approval granted by an NHS Research Ethics Committee in April 2021. REC reference is 21/SC/0080. IRAS project ID is 281193. Under this ethical approval, written informed consent was not required as the study utilized anonymized routine care data. The data was made available by Portsmouth Hospital University NHS Trust under a data sharing agreement.

## Results

The prevalence of modified HFRS was 59.3% for low frailty risk, 25.2% and 15.5% for intermediate and high, respectively. The characteristics and outcomes of patients are presented in Table 2. The mean age for patients was 67.8 years in group A (SOS codes-present), 75.0 years for patients in group B (NEWS≥7), 76.1 years for patients in group C (SOS codes-present and NEWS≥7), 62.3 years in group D (SOS codes-absent and NEWS<7), 74.0 years in group E (NEWS≥5), 75.3 years in group F (SOS codes-present and NEWS≥5), and 61.9 years in group G (SOS codes-absent and NEWS<5). About 53% of patients in sepsis-risk-positive groups (A, B, C, E, and F) were female, and about 51.8% for the sepsis-risk-negative groups (D and G). The mean modified Hospital Frailty Risk Score (HFRS), Charlson Comorbidity Index (CCI), and length of stay (LOS) were higher in patients in sepsis-risk-positive groups (A, B, C, E and F) compared to patients in sepsis-risk-negative groups (D and G).

**Table 2:** Patients’ characteristics and outcomes among patients at sepsis-risk-positive groups (A, B, C, E, and F) and the sepsis-risk-negative group (D and G) based on SOS codes and NEWS.

	Group A: SOS codes-present (n = 132,786)	Group B: NEWS≥7 (n = 13,007)	Group C: SOS codes-present with NEWS≥7 (n = 9,572)	Group D: SOS codes-absent with NEWS<7 (n = 170,216)	Group E: NEWS≥5 (n = 34,978)	Group F: SOS codes-present with NEWS≥5 (n = 23,787)	Group G: SOS codes-absent with NEWS<5 (n = 162,460)
**Age in years**							
Mean (SD)	67.8 (20.7)	75.0 (15.5)	76.1 (14.6)	62.3 (21.1)	74.0 (16.3)	75.3 (15.4)	61.9 (21.2)
Median (IQR)	73.0 (55.0-84.0)	78.0 (67.0-86.0)	79.0 (69.0-87.0)	66.0 (47.0-80.0)	78.0 (66.0-86.0)	78.0 (68.0-86.0)	66.0 (46.0-80.0)
P value	<0.001	<0.001	<0.001		<0.001	<0.001	
**Gender**							
Female No· (%)	71065 (53.6%)	7029 (54.0%)	5073 (52.9%)	88350 (51.9%)	18970 (54.2%)	12759 (53.6%)	84095 (51.8%)
Male No· (%)	61721 (46.4%)	5978 (46.0%)	4499 (47.1%)	81866 (48.1%)	16008 (45.8%)	11028 (46.4%)	78365 (48.2%)
P value	<0.001	<0.001	0.03		<0.001	<0.001	
**CCI**							
Mean (SD)	6.8 (10.5)	8.4 (10.5)	8.6 (11.4)	4.4 (8.4)	7.9 (10.5)	8.3 (11.4)	4.3 (8.3)
Median (IQR)	0.0(0.0-11.0)	4.0 (0.0-14.0)	4.0 (0.0-14.0)	0.0 (0.0-5.0)	3.0 (0.0-12.0)	3.0 (0.0-14.0)	0.0 (0.0-5.0)
P value	<0.001	<0.001	<0.001		<0.001	<0.001	
**Modified HFRS points**							
Mean (SD)	10.3 (10.7)	11.2 (10.0)	12.1 (10.1)	5.3 (7.6)	10.7 (10.1)	12.0 (10.3)	5.2 (7.3)
Median (IQR)	6.9 (2.1-15.5)	8.5 (3.6-16.2)	9.5 (4.5-17.3)	2.3 (0.4-7.2)	7.9 (3.0-16.0)	9.3 (4.0-17.5)	2.2 (0.3-7.0)
P value	<0.001	<0.001	<0.001		<0.001	<0.001	
**Modified HFRS categories No. (%)**							
Low (score<5)	54442 (41.0%)	4203 (32.3%)	2613 (27.3%)	112343(66.0%)	12489 (35.7%)	7076 (29.7%)	108318(66.7%)
Intermediate (score 5–15)	45938 (34.6%)	5333 (41.0%)	4087 (42.7%)	42043 (24.7%)	13399 (38.3%)	9586 (40.3%)	39477 (24.3%)
High (score>15)	32406 (24.4%)	3471 (26.7%)	2872 (30.0%)	15830 (9.3%)	9090 (26.0%)	7125 (30.0%)	14665 (9.0%)
P value	<0.001	<0.001	<0.001		<0.001	<0.001	
**LOS in days**							
Mean (SD)	11.1 (17.3)	11.3 (14.8)	12.7 (15.9)	4.8 (9.0)	11.2 (15.5)	13.1 (17.3)	4.7 (9.0)
Median (IQR)	5.0 (2.0-13.0)	7.0 (3.0-14.0)	7.0 (4.0-16.0)	2.0 (1.0-5.0)	6.0 (3.0-13.0)	7.0 (4.0-16.0)	2.0 (1.0-5.0)
P value	<0.001	<0.001	<0.001		<0.001	<0.001	
**Death of this discharge**							
No. (%)	11535 (8.7%)	3013 (23.2%)	2388 (25.0%)	3221 (1.9%)	5681 (16.2%)	4388 (18.4%)	2553 (1.6%)
P value	<0.001	<0.001	<0.001		<0.001	<0.001	

**SOS:** Suspicion of Sepsis**; NEWS:** National Early Warning Score**; HFRS:** Hospital frailty risk score**; CCI:** Charlson Comorbidity Index.

**P values** were from comparing sepsis-risk-positive groups with the sepsis-risk-negative groups**; P-values**<0.05 were considered statistically significant.

Over 58% of patients were categorised as having intermediate or high risk of frailty in sepsis-risk-positive groups: A (SOS codes-present), B (NEWS≥7), C (SOS codes-present and NEWS≥7), E (NEWS≥5), and F (SOS codes-present and NEWS≥5); while about 33.0% in the sepsis-risk-negative groups: D (SOS codes-absent and NEWS<7) and G (SOS codes-absent and NEWS<5). The proportion of in-hospital mortality at discharge was higher in sepsis-risk-positive groups (8.7%, 23.2%, 25.0%, 16.2%, and 18.4% in group A, B, C, E, and F respectively) than in sepsis-risk-negative groups (1.9% in group D and 1.6% in group G). Patients at sepsis-risk-positive groups: A (SOS codes-present), B (NEWS≥7), C (SOS codes-present and NEWS≥7), E (NEWS≥5), and F (SOS codes-present and NEWS≥5) were more likely to die in hospital compared to the sepsis-risk-negative groups: D (SOS codes-absent and NEWS<7) and G (SOS codes-absent and NEWS<5). All sepsis-risk-positive groups (A, B, C, E, and F) were significantly associated with increased risk of frailty (modified HFRS), longer LOS and in-hospital mortality (P < 0.001) compared with the sepsis-risk-negative (groups D and G).

**Fig 2:**
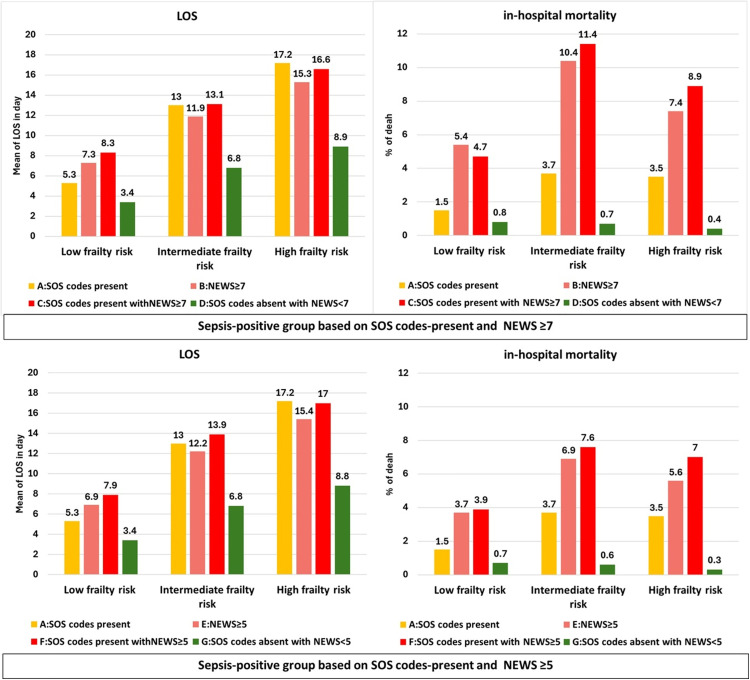
Association between modified HFRS and poor health outcomes for patients at risk of sepsis-risk-positive or sepsis-risk-negative.

Association between modified HFRS categories and poor health outcomes for patients at sepsis-risk-positive and the sepsis-risk-negative groups are presented in Fig 2. Overall, patients with intermediate or high frailty risk scores had a longer length of stay and a higher proportion of in-hospital mortality compared to the low frailty risk group. Patients at sepsis-risk-positive groups: A (SOS codes-present), B (NEWS≥7), C (SOS codes-present and NEWS≥7), E (NEWS≥5), and F (SOS codes-present and NEWS≥5) stayed-longer in hospital and had a higher proportion of in-hospital mortality than the sepsis-risk-negative groups: D (SOS codes-absent and NEWS<7) and G (SOS codes-absent and NEWS<5) with P value<0.001. Moreover, patients at sepsis-risk-positive who had intermediate or high frailty risk scores had a longer length of stay and a higher proportion of in-hospital mortality compared to the sepsis-risk-negative groups with low frailty risk scores (P < 0.001). However, sepsis-risk-positive groups based on NEWS≥7 (groups: B (NEWS≥7) and C (SOS codes-present and NEWS≥7) were more sensitive with in-hospital mortality than NEWS≥5 (groups: E (NEWS≥5) and F (SOS codes-present and NEWS≥5).

Results of the logistic regression with interaction models for modified HFRS and poor outcomes among patients with a probability of sepsis (based on SOS codes and NEWS≥7) are presented in Table 3. Patients with higher frailty risk (intermediate or high modified HFRS) and sepsis-risk-positive groups: A (SOS codes-present), B (NEWS≥7) and C (SOS codes-present and NEWS≥7) were significantly more likely to have a longer length of stay and a higher risk of mortality compared to low frailty risk group and the sepsis-risk-negative group D (SOS codes-absent and NEWS<7). The odds ratios ranged from 2.0 to 19.4 for LOS and from 2.0 to 5.1 for in-hospital mortality.

**Table 3:** Results of logistic regression with interaction models for modified HFRS and poor outcomes among patient with the probability of sepsis (based on SOS codes and NEWS≥7).

	Group A: SOS code-present			Group B: NEWS≥7			Group C: SOS codes-present with NEWS≥7		
outcomes	Odds Ratio (95% CI)			Odds Ratio (95% CI)			Odds Ratio (95% CI)		
	Interaction HFRS: sepsis-risk-positive (P- value)			Interaction HFRS: sepsis-risk-positive (P- value)			Interaction HFRS: sepsis-risk-positive (P- value)		
	Low frailty risk and SOS codes-absent	Intermediate frailty risk	High frailty risk	Low frailty risk and NEWS<7	Intermediate frailty risk	High frailty risk	Low frailty risk, SOS codes-absent and NEWS<7	Intermediate frailty risk	High frailty risk
**LOS > 3-day**	Reference	2.55 (2.5-2.65)	3.1 (3.0-3.6)	Reference	2.99 (2.7-3.2)	5.1 (4.9-5.5)	Reference	2.0 (1.9-2.5)	3.0 (2.9-3.3)
		P < 0.001	P < 0.001		P < 0.001	P < 0.001		P < 0.001	P < 0.001
**LOS > 7-day**	Reference	3.0 (2.9-3.4)	4.9 (4.7-5.3)	Reference	3.5 (3.0-3.9)	6.9 (6.7-7.3)	Reference	2.8 (2.6-3.1)	4.1 (4.0-4.7)
		P < 0.001	P < 0.001		P < 0.001	P < 0.001		P < 0.001	P < 0.001
**LOS > 10-day**	Reference	3.2 (3.6-3.8)	5.2 (5.0-5.8)	Reference	4.0 (3.9-4.2)	7.5 (7.0-8.1)	Reference	3.0 (2.9-3.4)	4.8 (4.6-5.3)
		P < 0.001	P < 0.001		P < 0.001	P < 0.001		P < 0.001	P < 0.001
**LOS > 14-day**	Reference	3.99 (3.9-4.0)	6.1 (5.9-6.8)	Reference	4.4 (4.0-4.7)	8.2 (8.0-8.9)	Reference	3.4 (3.0-3.8)	5.4 (5.0-6.1)
		P < 0.001	P < 0.001		P < 0.001	P < 0.001		P < 0.001	P < 0.001
**LOS > 21-day**	Reference	4.2 (4.0-4.5)	7.2 (7.0-8.1)	Reference	4.8 (4.6-5.2)	9.2 (8.9-10.1)	Reference	3.8 (3.6-4.3)	6.2 (6.0-7.1)
		P < 0.001	P < 0.001		P < 0.001	P < 0.001		P < 0.001	P < 0.001
**LOS > 30-day**	Reference	4.3 (4.1-5.0)	8.1 (7.8-9.4)	Reference	5.0 (4.9-5.5)	10.0 (9.7-10.9)	Reference	4.0 (3.9-4.6)	7.0 (6.8-8.1)
		P < 0.001	P < 0.001		P < 0.001	P < 0.001		P < 0.001	P < 0.001
**LOS > 45-day**	Reference	5.0 (4.7-5.8)	8.3 (7.6-10.0)	Reference	5.1 (5.0-6.0)	10.5 (10.0-11.7)	Reference	4.1 (3.9-5.2)	7.1 (6.5-8.6)
		P < 0.001	P < 0.001		P < 0.001	P < 0.001		P < 0.001	P < 0.001
**LOS > 60-day**	Reference	5.1 (4.5-6.4)	7.3 (6.4-9.6)	Reference	6.0 (5.6-6.9)	11.0 (10.2-12.5)	Reference	4.3 (3.9-5.7)	6.3 (5.5-8.3)
		P < 0.001	P < 0.001		P < 0.001	P < 0.001		P < 0.001	P < 0.001
**LOS > 90-day**	Reference	6.9 (4.8-11.0)	9.4 (6.3-15.7)	Reference	10.9 (9.3-13.6)	19.4 (11.0-20.4)	Reference	6.1 (4.3-10.0)	8.2 (5.4-10.4)
		P < 0.001	P < 0.001		P < 0.001	P < 0.001		P < 0.001	P < 0.001
**3 day-mortality**	Reference	2.0 (2.0-2.4)	2.1 (1.9-2.6)	Reference	2.3 (2.0-2.7)	2.4 (2.0-3.1)	Reference	2.0 (1.9-2.5)	2.0 (1.9-2.8)
		P < 0.001	P < 0.001		P < 0.001	P < 0.001		P < 0.001	P < 0.001
**7 day-mortality**	Reference	2.2 (2.3-2.7)	2.2 (2.0-2.8)	Reference	2.7 (2.5-3.1)	3.0 (2.9-3.6)	Reference	2.1 (2.0-2.7)	2.2 (2.0-2.8)
		P < 0.001	P < 0.001		P < 0.001	P < 0.001		P < 0.001	P < 0.001
**10 day-mortality**	Reference	2.3 (2.4-2.8)	2.4 (2.0-3.0)	Reference	2.9 (2.7-3.2)	3.1 (2.9-3.8)	Reference	2.2 (2.0-2.7)	2.4 (2.0-3.0)
		P < 0.001	P < 0.001		P < 0.001	P < 0.001		P < 0.001	P < 0.001
**14 day-mortality**	Reference	2.4 (2.5-2.9)	2.9 (2.3-3.1)	Reference	3.0 (2.9-3.4)	3.4 (3.0-4.1)	Reference	2.3 (2.1-2.8)	2.5 (2.1-3.1)
		P < 0.001	P < 0.001		P < 0.001	P < 0.001		P < 0.001	P < 0.001
**30 day-mortality**	Reference	2.5 (2.6-3.0)	2.99 (2.5-3.4)	Reference	3.1 (2.9-3.7)	4.4 (4.0-5.0)	Reference	2.4 (2.2-3.0)	2.9 (2.7-3.4)
		P < 0.001	P < 0.001		P < 0.001	P < 0.001		P < 0.001	P < 0.001
**60 day-mortality**	Reference	2.5 (2.7-3.0)	3.0 (2.8-3.5)	Reference	3.4 (3.0-3.9)	5.0 (4.7-5.6)	Reference	2.5 (2.3-3.0)	3.0 (2.8-3.6)
		P < 0.001	P < 0.001		P < 0.001	P < 0.001		P < 0.001	P < 0.001
**90 day-mortality**	Reference	2.6 (2.7-3.0)	3.1 (2.8-3.5)	Reference	3.5 (3.0-4.0)	5.1 (4.8-5.7)	Reference	2.5 (2.3-3.0)	3.0 (2.8-3.6)
		P < 0.001	P < 0.001		P < 0.001	P < 0.001		P < 0.001	P < 0.001
**6month-mortality**	Reference	2.6 (2.7-3.0)	3.1 (2.8-3.5)	Reference	3.5 (3.0-4.0)	5.1 (4.8-5.8)	Reference	2.5 (2.3-3.0)	3.0 (2.8-3.6)
		P < 0.001	P < 0.001		P < 0.001	P < 0.001		P < 0.001	P < 0.001

The interaction between modified HFRS and sepsis-risk-positive was statistically significant for all LOS and for all in-hospital mortality periods (P < 0.001), indicating that the effect of frailty risk (modified HFRS) on LOS or in-hospital mortality differed between patients with sepsis-risk-positive and those with sepsis-risk-negative. In addition, this was more pronounced across all LOS and in-hospital mortality periods in those with higher frailty risk scores compared to intermediate frailty risk scores.

The c-statistic values with 95% confidence intervals (CIs) for the interaction models between modified HFRS and sepsis risk-positive status (SOS codes-present, NEWS≥7, or SOS codes-present plus NEWS≥7) for each period of LOS and in-hospital mortality are presented in S1 Table. HFRS had acceptable discrimination for all periods of poor outcomes and for all sepsis-risk-positive groups; the c-statistic ranged from 0.639 to 0.799 for LOS, and from 0.616 to 0.725 for in-hospital mortality. Additionally, Group A (SOS codes-present) was superior to the other models for long LOS, with c-statistics ranging from 0.696 to 0.799, while Group C (SOS codes-present and NEWS≥7) was superior to the other models for in-hospital mortality, with c-statistics ranging from 0.707 to 0.725.

Further analyses were performed for patients with a probability of sepsis based on SOS codes and NEWS≥5. We obtained the same study results; odds ratios ranged from 2.0 to 12.3 for LOS and from 1.9 to 4.8 for in-hospital mortality, as shown in S2 Table.

Additional analyses were performed using the original HFRS by including the current “index” admission into the HFRS calculation, and we obtained similar results to the modified HFRS, as shown in S3 Table.

Further, we performed additional analyses for the logistic regression with interaction models for modified HFRS and poor outcomes among patients with a probability of sepsis (based on SOS codes and NEWS≥7) according to three age groups (patients aged from 16–49, from 50–74, and 75 and above), as shown in S4(a-c) Tables. We found that in all three age groups, patients with higher frailty risk and sepsis-risk-positive groups: A (SOS codes-present), B (NEWS≥7) and C (SOS codes-present and NEWS≥7) were significantly more likely to have a longer length of stay and a higher risk of mortality compared to the low frailty risk group and the sepsis-risk-negative group D (SOS codes-absent and NEWS<7). These age groups’ results were consistent with the findings across all adult ages. Moreover, the results of the three groups were consistent with each other. Odds ratios for patients aged 16–49 years ranged from 1.8–11.4 for LOS and 1.3–3.5 for mortality; for patients aged 50–74 years, they ranged from 3.0–15.3 for LOS and 1.6–4.0 for mortality; and for patients aged ≥75 years, they ranged from 2.1–13.8 for LOS and 1.3–3.6 for mortality.

The predicted probability effect of Suspicion of Sepsis (SOS) and the National Early Warning Score (NEWS≥7) on prolonged length of stay (LOS) according to HFRS categories is presented in S1 Fig. In general, the sepsis-risk-positive groups: A (SOS codes-present), B (NEWS≥7) and C (SOS codes-present and NEWS≥7) had a greater effect on the predictive power of HFRS in predicting long LOS compared to the sepsis-risk-negative group D (SOS codes-absent and NEWS<7). This indicates a strong association between risk of frailty (HFRS) and the sepsis-risk-positive in predicting hospital stay. However, the influence of the sepsis-risk-positive decreases with longer LOS.

The predicted probability effect of Suspicion of Sepsis (SOS codes) and National Early Warning Score (NEWS) on in-hospital mortality according to modified HFRS categories is presented in S2 Fig. Overall, the sepsis-risk-positive groups: A (SOS codes-present), B (NEWS≥7) and C (SOS codes-present and NEWS≥7) had a greater predicted probability effect on the modified HFRS to predict in-hospital mortality compared to the sepsis-risk-negative group D (SOS codes-absent and NEWS<7). The influence of SOS codes-present or NEWS≥7 increases with in-hospital mortality, demonstrating a strong association between risk of frailty (modified HFRS) and sepsis-risk-positive in predicting in-hospital mortality.

Additional analyses were performed to measure predicted probability for patients with a probability of sepsis based on SOS codes and NEWS≥5 for LOS and in-hospital mortality. We obtained the same study results; as shown in S3 and S4 Figs. However, sepsis-risk-positive groups based on NEWS≥7 performed better than NEWS≥5 groups for in-hospital mortality.

Additional analyses were performed using Restricted Cubic Splines (RCS) to test the non-linear relationship between continuous modified HFRS and all periods of prolonged LOS and in-hospital mortality, as presented in Table 4. The relationship between modified HFRS and length of stay or in-hospital mortality was significantly non-linear across all time periods of LOS and in-hospital mortality (P < 0.001). This indicates that the effect of modified HFRS on LOS or in-hospital mortality differs depending on the score level.

**Table 4:** Results of Restricted Cubic Splines (RCS) models for modified HFRS and poor outcomes.

Outcomes	Overall model	Non-linearity test	Effect estimate
	F (P-value)	F (P-value)	Effect (95% CI)
**Length of stay**			
LOS > 3-day	21358.3 (P < 0.0001)	8662.2 (P < 0.0001)	0.335 (0.331-0.338)
LOS > 7-day	21094.2 (P < 0.0001)	6413.5 (P < 0.0001)	0.275 (0.272-0.278)
LOS > 10-day	18164.2 (P < 0.0001)	4906.0 (P < 0.0001)	0.226 (0.223-0.228)
LOS > 14-day	14444.9 (P < 0.0001)	3560.6 (P < 0.0001)	0.174 (0.171-0.176)
LOS > 21-day	9669.6 (P < 0.0001)	2058.5 (P < 0.0001)	0.112 (0.110-0.114)
LOS > 30-day	6025.4 (P < 0.0001)	1154.4 (P < 0.0001)	0.068 (0.067-0.070)
LOS > 45-day	2939.6 (P < 0.0001)	588.6 (P < 0.0001)	0.034 (0.033-0.035)
LOS > 60-day	1509.8 (P < 0.0001)	332.2 (P < 0.0001)	0.019 (0.017-0.02)
LOS > 90-day	498.3 (P < 0.0001)	194.9 (P < 0.0001)	0.012 (0.011-0.014)
**In-hospital mortality**			
3 day-mortality	528.4 (P < 0.0001)	379.1 (P < 0.0001)	0.016 (0.015-0.017)
7 day-mortality	1087.3 (P < 0.0001)	697.9 (P < 0.0001)	0.027 (0.026-0.028)
10 day-mortality	1423.9 (P < 0.0001)	889.8 (P < 0.0001)	0.034 (0.033-0.035)
14 day-mortality	1785.4 (P < 0.0001)	1048.4 (P < 0.0001)	0.041 (0.039-0.042)
30 day-mortality	2704.7 (P < 0.0001)	1325.9 (P < 0.0001)	0.054 (0.053-0.056)
60 day-mortality	3213.1 (P < 0.0001)	1398.1 (P < 0.0001)	0.060 (0.058-0.061)
90 day-mortality	3313.4 (P < 0.0001)	1411.4 (P < 0.0001)	0.061 (0.059-0.063)
6month-mortality	3355.5 (P < 0.0001)	1411.5 (P < 0.0001)	0.062 (0.060-0.063)

**Fig 3:**
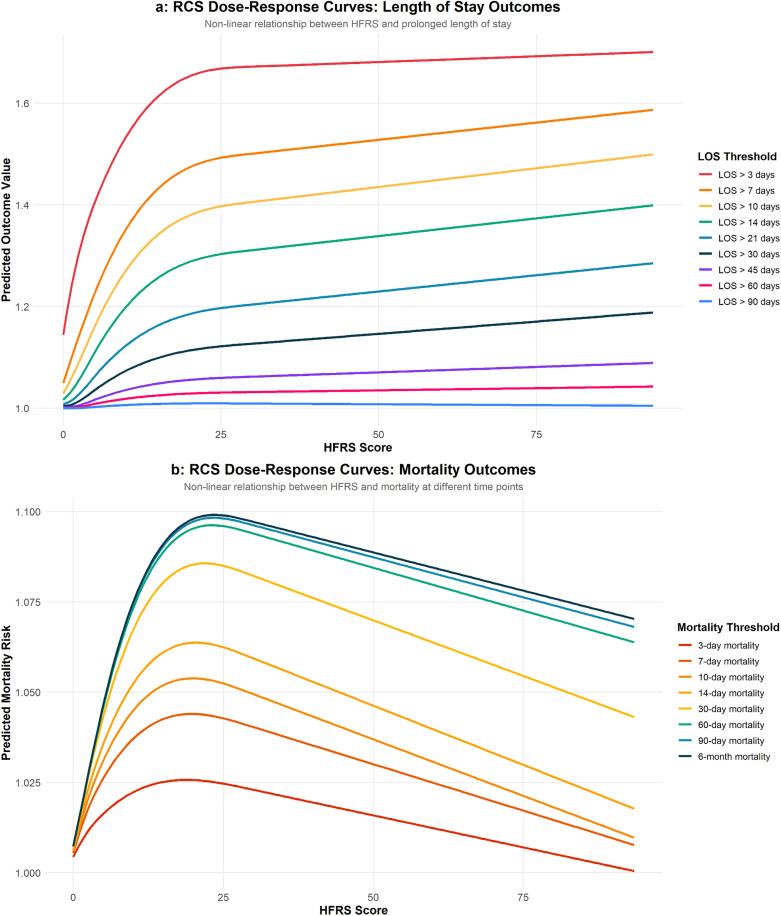
RCS dose-response curves between modified HFRS and poor outcome: (a) is LOS; (b) is mortality.

The dose-response curves demonstrated significant non-linear relationships between modified HFRS and all periods of LOS or in-hospital mortality, as shown in Fig 3.

## Discussion

In this large group of non-electively admitted hospital patients of all adult ages we found that those at high risk of sepsis (SOS codes present and/ NEWS 7 or more) were more likely to have risk of frailty (modified HFRS intermediate/severe), stay in hospital longer and had increased mortality rates 1 in 4 with SOS codes positive and a high NEWS died during the index admission. The probability of high risk of sepsis increased in those with risk of frailty, and those with risk of frailty and high risk of sepsis stayed longer in hospital and were more likely to die than those without. To our knowledge, this is the first study of HFRS and risk of sepsis in a broad group of hospitalized non-electively admitted adults. However, our findings are in keeping with those of others that frailty and sepsis are associated with adverse outcomes and increased healthcare utilisation [23,25,26,39,40].

Additionally, the predicted probability effect of sepsis-risk-positive was higher than that of the sepsis-risk-negative on the predictive power of the modified HFRS for all LOS and in-hospital mortality periods suggesting that risk of frailty may represent a risk factor for sepsis-risk-positive. This aligns with other studies of frailty, using the HFRS, and sepsis and infection risk in narrower cohorts of patients for example in those undergoing kidney transplantation, a modified HFRS predicted the risk of developing a severe infection [41]. HFRS was also predictive for the risk of developing postoperative sepsis in a group of older people undergoing surgery [19].

The interaction effect of modified HFRS and risk of sepsis on in-hospital mortality was most marked at 7 or more days LOS. It is interesting that the mortality association was similar in both the intermediate and high frailty risk groups throughout but with intermediate frailty risk groups having higher predicted probabilities than those with high frailty risk. A possible explanation is that those with severe frailty risk might be more easily recognised by clinicians than the intermediate frailty risk level group and thus treatments and approaches to care, including setting, are tailored accordingly.

The utility of the HFRS as an identification tool in clinical practice is limited by the inclusion of coding for the current admission which is typically not available until after discharge. In the setting of an Emergency Department, a study of a modified HFRS (which did not include the index admission coding) in older adults admitted to hospital found that this was able to identify non-frail patients but was less good for those with severe frailty risk [42]. We found that a modified HFRS performed similarly to the original for adults of all ages, indicating its potential use as an identification tool at the point of admission based on prior codes.

SOS codes were developed as a tool for monitoring cohorts at increased risk of sepsis rather than as a clinical tool. We found their presence increased with risk of frailty and were associated with increased mortality and length of stay when present. However, the presence of an elevated NEWS score was more strongly associated with increased mortality and length of stay and the combination of elevated NEWS scores and SOS codes did not appear to increase the association.

This perhaps suggests that once there is severe physiological derangement the possible cause of the derangement becomes less relevant.

We found fair to good c-statistics for the modified HFRS in those who were risk of sepsis-positive. This was consistently good (above 0.7) for those with LOS over 7 days and for mortality at all stages in those with SOS codes present and a NEWS of 7 or more. The c- statistics are higher than those in the initial un-modified HFRS development [17]. Further research would be needed to confirm this finding in other cohorts. We recognise that utility at an individual patient level would be limited in the context of sepsis, as the knowledge of the sepsis codes is not known at the point of admission. Further analyses were performed to measure interaction and prediction for patients with a probability of sepsis based on SOS codes and NEWS≥5 for LOS and in-hospital mortality. We found that sepsis-risk-positive groups based on NEWS≥7 had more effect on modified HFRS in predicting in-hospital mortality than NEWS≥5 groups. This is in keeping with other studies that found NEWS≥7 was more sensitive in identifying patients with suspected sepsis and was strongly associated with the outcome of sepsis [35,37,38,43].

Furthermore, additional analyses stratified by age (16–49, 50–74, and ≥75 years) demonstrated consistent associations across three age groups. Patients with higher frailty risk and sepsis-risk-positive were significantly more likely to have a longer hospital stay and higher in-hospital mortality compared with patients at low frailty risk and sepsis-risk-negative. Moreover, the results among the three groups were consistent with each other. These findings align with a recent study reporting that age does not influence the ability of the HFRS to predict LOS across all age groups [30]. Frailty is not usually routinely considered in those under 65 years during a hospital admission so identifying tools that can be applied from routine clinical work may have utility in highlighting those at potential additional risk.

The additional analyses conducted using Restricted Cubic Splines (RCS) demonstrate a significant nonlinear relationship between modified HFRS and both length of stay (LOS) and in-hospital mortality across all time periods (P < 0.001). This finding suggests that the impact of frailty risk, as measured by the modified HFRS, on these clinical outcomes is not constant but changes depending on the level of the HFRS score. Furthermore, the dose-response curves presented in S3 Fig and S4 Fig indicate that interventions may be more effective for patients with intermediate or high levels of frailty risk, where the greatest changes in risk are observed.

The study has some strengths. We used a large population of patients including all adult ages. Also, we used multiple cut-offs for LOS and in-hospital mortality as study outcomes, which might help to explore the association between patient complexity and longer LOS and in-hospital mortality among patients at risk of frailty and sepsis-risk-positive. The study used 2 indicators (NEWS and SOS codes) to identify patients at risk of sepsis.

The findings showed that the sepsis-risk-positive and a higher score of modified HFRS were both associated with longer LOS and in-hospital mortality. However, our study has some limitations. Although we used a large data set, it is from one hospital, so further research is needed to explore whether the results are replicated in different settings of care. Risk of frailty was determined by the HFRS. This has been widely used and validated by others but may not capture the same cohorts of patients as other measures such as the Clinical Frailty Scale [44], as the HFRS uses ICD-10 diagnostic codes related to frailty rather than direct clinical assessment. Whilst HFRS is easy to calculate without additional work for clinical staff, it does not fully reflect the physiological vulnerability or functional decline that defines frailty. However, the HFRS may serve as a useful tool for frailty risk stratification. Our data had relatively few uncoded episodes compared to the original validation HFRS cohort [17], any approach based on coding relies on this being accurate. Additionally, the requirement for two years of preceding hospital data to calculate the HFRS resulted in the exclusion of patients without previous admissions, who may represent a healthier cohort with a lower frailty risk. This exclusion may limit the generalisability of the findings to this potentially healthier population.

We acknowledge that the use of a high NEWS2 score identifies not only those with risk of sepsis but also those with physiological derangement due to other conditions. It is also possible that some older people with frailty and sepsis did not have a high NEWS2 score on admission [45]. Because our analysis defined sepsis risk based only on the NEWS2 score rather than a confirmed infection, some patients without sepsis may have been misclassified as high risk, while others with sepsis but lower NEWS2 scores may have been missed. This methodological limitation may have influenced the accuracy of sepsis-related findings in our study.

The HFRS is not usually used in clinical care but has been suggested as a relevant tool to identify cohorts of patients at increased risk [46]. The SOS similarly uses routine coding data and has been suggested as a tool for monitoring the impact of sepsis improvement programmes. Given that frailty is strongly associated with both the development of sepsis and of poor outcomes from sepsis there is potential for utilisation of HFRS alongside other monitoring tools, in research on sepsis and to identify cohorts of patients in hospitals who are at increased risk of adverse outcomes [33].

## Conclusion

There is a strong association between risk of frailty (as measured using the Hospital Frailty Risk Score) and sepsis-risk-positive (using SOS codes and physiological parameters) for the outcomes of mortality and length of stay, and we have demonstrated that this applies to hospitalised adults of all ages. Understanding that those with frailty, whatever their age, are at increased risk both of developing sepsis and of adverse outcomes if they develop it, is important for clinicians for identifying those at increased risk, rapid implementation of appropriate management – whether that is an intensive treatment approach or, where appropriate and after holistic clinical review, consideration of symptom-based care. The modified-HFRS offers the potential for operational use. Future research could focus on whether these findings are replicated in other hospitals and care settings, the utility of HFRS or modified-HFRS as a routine frailty risk identification tool in cohorts at increased risk of sepsis development who may benefit from enhanced care, or in sepsis research and improvement monitoring.

## Supporting information

S1 Table C-statistics with 95% CI for interaction models between modified HFRS and the probability of sepsis (based on SOS codes and NEWS≥7) for all period of poor outcomes. (DOCX)

S2 Table Results of logistic regression with interaction models for modified HFRS and poor outcomes among patient with the probability of sepsis (based on SOS codes and NEWS≥5). (DOCX)

S3 Table Results of logistic regression with interaction models for original HFRS and poor outcomes among patient with the probability of sepsis (based on SOS codes and NEWS≥7). (DOCX)

S4 Table Results of logistic regression with interaction models for modified HFRS and poor outcomes among patient with the probability of sepsis (based on SOS codes and NEWS≥7) according to three age groups (patients aged 16–49, 50–74, and ≥75). (DOCX)

S1 Fig Interaction effect of modified HFRS and risk of sepsis (based on SOS codes and NEWS≥7) on LOS. (TIF)

S2 Fig Interaction effect of modified HFRS and risk of sepsis (based on SOS codes and NEWS≥7) on in-hospital mortality. (TIF)

S3 Fig Interaction effect of modified HFRS and risk of sepsis (based on SOS codes and NEWS≥5) on LOS. (TIF)

S4 Fig Interaction effect of modified HFRS and risk of sepsis (based on SOS codes and NEWS≥5) on in-hospital mortality. (TIF)
