# Supplementary material for: Association between hospital frailty risk score, risk of sepsis and adverse outcomes across all adult ages
Source: PLoS One. 2026 Feb 13;21(2):e0342790. doi: 10.1371/journal.pone.0342790 (PMC12904455; doi:10.1371/journal.pone.0342790)
Supplement: S1 Table — (DOCX) [file pone.0342790.s001.docx]

S1 Table. C-statistics with 95% CI for interaction models between modified HFRS and the probability of sepsis (based on SOS codes and NEWS≥7) for all period of poor outcomes

|  | **Group A: SOS code-present** | | **Group B: NEWS≥7** | | **Group C: SOS codes-present with NEWS≥7** | |
| --- | --- | --- | --- | --- | --- | --- |
|  | **C-statistics** | **95% CI** | **C-statistics** | **95% CI** | **C-statistics** | **95% CI** |
| **LOS>3-day** | 0.696 | (0.695-0.698) | 0.639 | (0.637-0.640) | 0.677 | (0.675-0.678) |
| **LOS>7-day** | 0.725 | (0.723-0.726) | 0.668 | (0.666-0.670) | 0.702 | (0.700-0.704) |
| **LOS>10-day** | 0.736 | (0.734-0.738) | 0.678 | (0.675-0.680) | 0.712 | (0.710-0.714) |
| **LOS>14-day** | 0.747 | (0.745-0.749) | 0.685 | (0.683-0.688) | 0.722 | (0.720-0.725) |
| **LOS>21-day** | 0.760 | (0.757-0.762) | 0.693 | (0.690-0.696) | 0.734 | (0.731-0.737) |
| **LOS>30-day** | 0.768 | (0.765-0.771) | 0.696 | (0.693-0.700) | 0.742 | (0.738-0.745) |
| **LOS>45-day** | 0.777 | (0.773-0.781) | 0.698 | (0.693-0.703) | 0.750 | (0.746-0.755) |
| **LOS>60-day** | 0.780 | (0.774-0.786) | 0.698 | (0.691-0.704) | 0.754 | (0.747-0.760) |
| **LOS>90-day** | 0.799 | (0.790-0.809) | 0.709 | (0.698-0.721) | 0.775 | (0.766-0.785) |
| **3 day-mortality** | 0.616 | (0.609-0.624) | 0.696 | (0.687-0.704) | 0.707 | (0.698-0.716) |
| **7 day-mortality** | 0.643 | (0.637-0.648) | 0.685 | (0.678-0.691) | 0.704 | (0.698-0.711) |
| **10 day-mortality** | 0.655 | (0.650-0.660) | 0.682 | (0.676-0.687) | 0.705 | (0.74-0.711) |
| **14 day-mortality** | 0.665 | (0.661-0.670) | 0.679 | (0.674-0.684) | 0.706 | (0.701-0.711) |
| **30 day-mortality** | 0.688 | (0.684-0.692) | 0.683 | (0.679-0.687) | 0.716 | (0.711-0.76) |
| **60 day-mortality** | 0.700 | (0.696-0.704) | 0.687 | (0.683-0.691) | 0.723 | (0.719-0.727) |
| **90 day-mortality** | 0.702 | (0.698-0.706) | 0.688 | (0.683-0.692) | 0.724 | (0.76-0.728) |
| **6month-mortality** | 0.703 | (0.699-0.707) | 0.688 | (0.684-0.692) | 0.725 | (0.721-0.728) |
