# Supplementary material for: Association between hospital frailty risk score, risk of sepsis and adverse outcomes across all adult ages
Source: PLoS One. 2026 Feb 13;21(2):e0342790. doi: 10.1371/journal.pone.0342790 (PMC12904455; doi:10.1371/journal.pone.0342790)
Supplement: S2 Table — (DOCX) [file pone.0342790.s002.docx]

**S2 Table.** **Results of logistic regression with interaction models for modified HFRS and poor outcomes among patient with the probability of sepsis (SOS codes and NEWS≥5)**

|  | **Group A: SOS code-present** | | | **Group E: NEWS≥5** | | | **Group F: SOS codes-present with NEWS≥5** | | |
| --- | --- | --- | --- | --- | --- | --- | --- | --- | --- |
| **outcomes** | **Odds Ratio (95% CI)** | | | **Odds Ratio (95% CI)** | | | **Odds Ratio (95% CI)** | | |
|  | **Interaction HFRS: sepsis-positive (P- value)** | | | **Interaction HFRS: sepsis-positive (P- value)** | | | **Interaction HFRS: sepsis-positive (P- value)** | | |
|  | **Low frailty risk and SOS codes-absent** | **Intermediate frailty risk** | **High frailty risk** | **Low frailty risk and NEWS<5** | **Intermediate frailty risk** | **High frailty risk** | **Low frailty risk, SOS codes-absent and NEWS<5** | **Intermediate frailty risk** | **High frailty risk** |
| **LOS> 3-day** | Reference | 2.0 (1.9-2.2) | 2.9 (2.7-2.9) | Reference | 2.5 (2.4-2.6) | 4.8 (4.7-4.9) | Reference | 2.0 (1.9-2.3) | 2.2 (2.0-2.5) |
|  |  | P < 0.001 | P < 0.001 |  | P < 0.001 | P < 0.001 |  | P < 0.001 | P < 0.001 |
| **LOS>7-day** | Reference | 2.8 (2.5-2.8) | 4.5 (4.0-4.9) | Reference | 3.3 (3.2-3.4) | 6.6 (6.5-6.9) | Reference | 2.0 (1.9-2.1) | 3.4 (3.2-3.8) |
|  |  | P < 0.001 | P < 0.001 |  | P < 0.001 | P < 0.001 |  | P < 0.001 | P < 0.001 |
| **LOS>10-day** | Reference | 3.2 (3.0-.3-3.6) | 5.1 (4.9-5.5) | Reference | 3.7 (3.6-3.9) | 7.0 (6.9-7.2) | Reference | 2.3 (2.2-2.5) | 4.0 (3.7-4.4) |
|  |  | P < 0.001 | P < 0.001 |  | P < 0.001 | P < 0.001 |  | P < 0.001 | P < 0.001 |
| **LOS>14-day** | Reference | 3.7 (3.5-3.8) | 6.0 (5.7-6.2) | Reference | 4.0 (3.8-4.2) | 7.7 (7.6-7.9) | Reference | 2.7 (2.5-2.9) | 4.6 (4.4-5.0) |
|  |  | P < 0.001 | P < 0.001 |  | P < 0.001 | P < 0.001 |  | P < 0.001 | P < 0.001 |
| **LOS>21-day** | Reference | 4.0 (3.9-4.4) | 7.0 (6.8-7.3) | Reference | 4.6 (4.4-4.7) | 8.6 (8.4-8.9) | Reference | 3.0 (2.8-3.3) | 6.0 (5.7-6.6) |
|  |  | P < 0.001 | P < 0.001 |  | P < 0.001 | P < 0.001 |  | P < 0.001 | P < 0.001 |
| **LOS>30-day** | Reference | 4.1 (4.0-4.8) | 8.1 (7.6-8.6) | Reference | 4.9 (4.8-5.2) | 9.5 (9.3-9.9) | Reference | 3.2 (2.9-3.5) | 6.0 (5.9-7.1) |
|  |  | P < 0.001 | P < 0.001 |  | P < 0.001 | P < 0.001 |  | P < 0.001 | P < 0.001 |
| **LOS>45-day** | Reference | 4.8 (4.1-5.5) | 8.1 (7.9-8.9) | Reference | 5.1 (4.9-5.4) | 10.0 (9.8-10.6) | Reference | 3.4 (2.9-3.9) | 6.3 (5.9-7.9) |
|  |  | P < 0.001 | P < 0.001 |  | P < 0.001 | P < 0.001 |  | P < 0.001 | P < 0.001 |
| **LOS>60-day** | Reference | 5.0 (4.7-5.9) | 7.1 (6.9-8.4) | Reference | 5.7 (5.5-6.0) | 10.5 (9.6-11.0) | Reference | 3.6(3.5-4.8) | 6.6 (5.2-8.0) |
|  |  | P < 0.001 | P < 0.001 |  | P < 0.001 | P < 0.001 |  | P < 0.001 | P < 0.001 |
| **LOS>90-day** | Reference | 6.7 (4.8-10.5) | 9.2 (6.2-13.0) | Reference | 7.1 (5.4-9.7) | 12.3 (11.5-15.4) | Reference | 5.4 (4.0-8.5) | 6.9 (5.0-9.3) |
|  |  | P < 0.001 | P < 0.001 |  | P < 0.001 | P < 0.001 |  | P < 0.001 | P < 0.001 |
| **3 day-mortality** | Reference | 2.0 (1.9-2.1) | 2.0 (1.8-2.1) | Reference | 1.9 (1.8-2.2) | 2.0 (1.8-2.3) | Reference | 2.0 (1.9-2.4) | 2.2 (2.1-2.3) |
|  |  | P < 0.001 | P < 0.001 |  | P < 0.001 | P < 0.001 |  | P < 0.001 | P < 0.001 |
| **7 day-mortality** | Reference | 2.0 (1.8-2.1) | 2.3 (2.1-2.6) | Reference | 2.1 (1.9-2.4) | 2.5 (2.2-2.7) | Reference | 2.0 (1.9-2.4) | 2.2 (2.1-2.4) |
|  |  | P < 0.001 | P < 0.001 |  | P < 0.001 | P < 0.001 |  | P < 0.001 | P < 0.001 |
| **10 day-mortality** | Reference | 2.1 (1.9-2.2) | 2.5 (2.3-2.7) | Reference | 2.3 (2.2-2.5) | 2.8 (2.6-3.1) | Reference | 2.1 (2.0-2.3) | 2.3 (2.2-2.5) |
|  |  | P < 0.001 | P < 0.001 |  | P < 0.001 | P < 0.001 |  | P < 0.001 | P < 0.001 |
| **14 day-mortality** | Reference | 2.2 (2.0-2.3) | 2.7 (2.5-2.9) | Reference | 2.5 (2.3-2.7) | 2.9 (2.6-3.2) | Reference | 2.1 (2.0-2.3) | 2.3 (2.2-2.5) |
|  |  | P < 0.001 | P < 0.001 |  | P < 0.001 | P < 0.001 |  | P < 0.001 | P < 0.001 |
| **30 day-mortality** | Reference | 2.3 (2.1-2.5) | 2.8 (2.6-3.1) | Reference | 2.8 (2.5-2.9) | 3.9 (3.8-4.2) | Reference | 2.2 (2.0-2.7) | 2.5 (2.3-3.0) |
|  |  | P < 0.001 | P < 0.001 |  | P < 0.001 | P < 0.001 |  | P < 0.001 | P < 0.001 |
| **60 day-mortality** | Reference | 2.4 (2.2-2.6) | 2.9 (2.8-3.1) | Reference | 3.0 (2.8-3.3) | 4.5 (4.3-5.0) | Reference | 2.2 (2.0-2.7) | 2.7 (2.3-3.0) |
|  |  | P < 0.001 | P < 0.001 |  | P < 0.001 | P < 0.001 |  | P < 0.001 | P < 0.001 |
| **90 day-mortality** | Reference | 2.5 (2.2-2.7) | 3.1 (2.8-3.3) | Reference | 3.1 (2.9-3.4) | 4.6 (4.6-4.9) | Reference | 2.5 (2.3-2.8) | 2.8 (2.4-2.9) |
|  |  | P < 0.001 | P < 0.001 |  | P < 0.001 | P < 0.001 |  | P < 0.001 | P < 0.001 |
| **6month-mortality** | Reference | 2.5 (2.2-2.7) | 3.1 (2.8-3.2) | Reference | 3.1 (2.9-3.4) | 4.8 (4.5-5.2) | Reference | 2.5 (2.3-2.8) | 3.0 (2.4-3.5) |
|  |  | P < 0.001 | P < 0.001 |  | P < 0.001 | P < 0.001 |  | P < 0.001 | P < 0.001 |
