# Supplementary material for: Association between hospital frailty risk score, risk of sepsis and adverse outcomes across all adult ages
Source: PLoS One. 2026 Feb 13;21(2):e0342790. doi: 10.1371/journal.pone.0342790 (PMC12904455; doi:10.1371/journal.pone.0342790)
Supplement: S3 Table — (DOCX) [file pone.0342790.s003.docx]

S3 Table. Results of logistic regression with interaction models for original HFRS* and poor outcomes among patient with the probability of sepsis (based on SOS codes and NEWS≥7)

|  | **Group A: SOS code-present** | | | **Group B: NEWS≥7** | | | **Group C: SOS codes-present with NEWS≥7** | | |
| --- | --- | --- | --- | --- | --- | --- | --- | --- | --- |
| **outcomes** | **Odds Ratio (95% CI)** | | | **Odds Ratio (95% CI)** | | | **Odds Ratio (95% CI)** | | |
|  | **Interaction HFRS: sepsis-risk-positive (P- value)** | | | **Interaction HFRS: sepsis-risk-positive (P- value)** | | | **Interaction HFRS: sepsis-risk-positive (P- value)** | | |
|  | **Low frailty risk and SOS codes-absent** | **Intermediate frailty risk** | **High frailty risk** | **Low frailty risk and NEWS<7** | **Intermediate frailty risk** | **High frailty risk** | **Low frailty risk, SOS codes-absent and NEWS<7** | **Intermediate frailty risk** | **High frailty risk** |
| **LOS> 3-day** | Reference | 2.6 (2.5-2.7) | 3.5 (3.4-3.6) | Reference | 3.1 (3.0-3.2) | 5.4 (5.3-5.5) | Reference | 2.4 (2.3-2.5) | 3.2 (3.1-3.3) |
|  |  | P < 0.001 | P < 0.001 |  | P < 0.001 | P < 0.001 |  | P < 0.001 | P < 0.001 |
| **LOS>7-day** | Reference | 3.3 (3.2-3.4) | 5.0 (4.8-5.1) | Reference | 3.8 (3.7-3.9) | 7.1 (7.0-7.3) | Reference | 3.0 (2.9-3.1) | 4.5 (4.3-4.7) |
|  |  | P < 0.001 | P < 0.001 |  | P < 0.001 | P < 0.001 |  | P < 0.001 | P < 0.001 |
| **LOS>10-day** | Reference | 3.7 (3.6-3.8) | 5.7 (5.5-6.0) | Reference | 4.1 (4.0-4.2) | 7.9 (7.7-8.1) | Reference | 3.3 (3.2-3.4) | 5.1 (4.9-5.3) |
|  |  | P < 0.001 | P < 0.001 |  | P < 0.001 | P < 0.001 |  | P < 0.001 | P < 0.001 |
| **LOS>14-day** | Reference | 4.1 (4.0-4.3) | 6.6 (6.3-6.9) | Reference | 4.6 (4.4-4.7) | 8.7 (8.4-8.9) | Reference | 3.7 (3.5-3.8) | 5.8 (5.5-6.1) |
|  |  | P < 0.001 | P < 0.001 |  | P < 0.001 | P < 0.001 |  | P < 0.001 | P < 0.001 |
| **LOS>21-day** | Reference | 4.6 (4.3-4.8) | 7.6 (7.2-8.1) | Reference | 5.0 (4.8-5.2) | 9.7 (9.4-10.1) | Reference | 4.0 (3.8-4.3) | 6.6 (6.2-7.1) |
|  |  | P < 0.001 | P < 0.001 |  | P < 0.001 | P < 0.001 |  | P < 0.001 | P < 0.001 |
| **LOS>30-day** | Reference | 4.9 (4.5-5.3) | 8.6 (7.9-9.4) | Reference | 5.2 (5.0-5.5) | 10.4 (9.9-10.9) | Reference | 4.2 (3.9-4.6) | 7.4 (6.8-8.1) |
|  |  | P < 0.001 | P < 0.001 |  | P < 0.001 | P < 0.001 |  | P < 0.001 | P < 0.001 |
| **LOS>45-day** | Reference | 5.2 (4.6-5.9) | 8.7 (7.6-10.0) | Reference | 5.5 (5.2-6.0) | 10.9 (10.2-11.7) | Reference | 4.5 (4.0-5.2) | 7.5 (6.5-8.6) |
|  |  | P < 0.001 | P < 0.001 |  | P < 0.001 | P < 0.001 |  | P < 0.001 | P < 0.001 |
| **LOS>60-day** | Reference | 5.4 (4.5-6.5) | 7.8 (6.4-9.6) | Reference | 6.2 (5.6-6.9) | 11.3 (10.2-12.5) | Reference | 4.7 (3.9-5.7) | 6.7 (5.5-8.3) |
|  |  | P < 0.001 | P < 0.001 |  | P < 0.001 | P < 0.001 |  | P < 0.001 | P < 0.001 |
| **LOS>90-day** | Reference | 7.2 (4.8-11.0) | 9.9 (6.3-15.7) | Reference | 11.2 (9.3-13.6) | 21.8 (18.0-26.4) | Reference | 6.5 (4.3-10.0) | 8.6 (5.4-10.4) |
|  |  | P < 0.001 | P < 0.001 |  | P < 0.001 | P < 0.001 |  | P < 0.001 | P < 0.001 |
| **3 day-mortality** | Reference | 2.2 (2.0-2.4) | 2.3 (2.1-2.6) | Reference | 2.5 (2.3-2.7) | 2.8 (2.5-3.1) | Reference | 2.2 (1.9-2.5) | 2.3 (2.0-2.8) |
|  |  | P < 0.001 | P < 0.001 |  | P < 0.001 | P < 0.001 |  | P < 0.001 | P < 0.001 |
| **7 day-mortality** | Reference | 2.5 (2.3-2.7) | 2.6 (2.3-2.8) | Reference | 2.9 (2.7-3.1) | 3.3 (3.1-3.6) | Reference | 2.4 (2.2-2.7) | 2.5 (2.2-2.8) |
|  |  | P < 0.001 | P < 0.001 |  | P < 0.001 | P < 0.001 |  | P < 0.001 | P < 0.001 |
| **10 day-mortality** | Reference | 2.6 (2.4-2.8) | 2.7 (2.5-3.0) | Reference | 3.0 (2.9-3.2) | 3.5 (3.3-3.8) | Reference | 2.5 (2.3-2.7) | 2.7 (2.4-3.0) |
|  |  | P < 0.001 | P < 0.001 |  | P < 0.001 | P < 0.001 |  | P < 0.001 | P < 0.001 |
| **14 day-mortality** | Reference | 2.7 (2.5-2.9) | 2.9 (2.6-3.1) | Reference | 3.2 (3.0-3.4) | 3.8 (3.6-4.1) | Reference | 2.6 (2.4-2.8) | 2.8 (2.5-3.1) |
|  |  | P < 0.001 | P < 0.001 |  | P < 0.001 | P < 0.001 |  | P < 0.001 | P < 0.001 |
| **30 day-mortality** | Reference | 2.8 (2.6-3.0) | 3.1 (2.9-3.4) | Reference | 3.5 (3.3-3.7) | 4.8 (4.5-5.0) | Reference | 2.7 (2.5-3.0) | 3.1 (2.8-3.4) |
|  |  | P < 0.001 | P < 0.001 |  | P < 0.001 | P < 0.001 |  | P < 0.001 | P < 0.001 |
| **60 day-mortality** | Reference | 2.8 (2.7-3.0) | 3.2 (3.0-3.5) | Reference | 3.7 (3.5-3.9) | 5.3 (5.1-5.6) | Reference | 2.8 (2.6-3.0) | 3.3 (3.0-3.6) |
|  |  | P < 0.001 | P < 0.001 |  | P < 0.001 | P < 0.001 |  | P < 0.001 | P < 0.001 |
| **90 day-mortality** | Reference | 2.9 (2.7-3.0) | 3.3 (3.0-3.5) | Reference | 3.8 (3.6-4.0) | 5.4 (5.2-5.7) | Reference | 2.8 (2.6-3.0) | 3.3 (3.0-3.6) |
|  |  | P < 0.001 | P < 0.001 |  | P < 0.001 | P < 0.001 |  | P < 0.001 | P < 0.001 |
| **6month-mortality** | Reference | 2.9 (2.7-3.0) | 3.3 (3.0-3.5) | Reference | 3.8 (3.6-4.0) | 5.5 (5.2-5.8) | Reference | 2.8 (2.6-3.0) | 3.3 (3.0-3.6) |
|  |  | P < 0.001 | P < 0.001 |  | P < 0.001 | P < 0.001 |  | P < 0.001 | P < 0.001 |

original HFRS*: calculate original HFRS by including the index admission into the HFRS calculation
