# Supplementary material for: Association between hospital frailty risk score, risk of sepsis and adverse outcomes across all adult ages
Source: PLoS One. 2026 Feb 13;21(2):e0342790. doi: 10.1371/journal.pone.0342790 (PMC12904455; doi:10.1371/journal.pone.0342790)
Supplement: S4 Table — (DOCX) [file pone.0342790.s004.docx]

S3-a Table. Results of logistic regression with interaction models for modified HFRS* and poor outcomes among patient with the probability of sepsis (based on SOS codes and NEWS≥7) For patients aged 16-49 years

|  | **Group A: SOS code-present** | | | **Group B: NEWS≥7** | | | **Group C: SOS codes-present with NEWS≥7** | | |
| --- | --- | --- | --- | --- | --- | --- | --- | --- | --- |
| **outcomes** | **Odds Ratio (95% CI)** | | | **Odds Ratio (95% CI)** | | | **Odds Ratio (95% CI)** | | |
|  | **Interaction HFRS: sepsis-risk-positive (P- value)** | | | **Interaction HFRS: sepsis-risk-positive (P- value)** | | | **Interaction HFRS: sepsis-risk-positive (P- value)** | | |
|  | **Low frailty risk and SOS codes-absent** | **Intermediate frailty risk** | **High frailty risk** | **Low frailty risk and NEWS<7** | **Intermediate frailty risk** | **High frailty risk** | **Low frailty risk, SOS codes-absent and NEWS<7** | **Intermediate frailty risk** | **High frailty risk** |
| **LOS> 3-day** | Reference | 1.8 (1.7-1.8) | 1.9 (1.8-2.0) | Reference | 2.4 (2.3-2.5) | 3.7 (3.6-3.8) | Reference | 1.9 (1.8-2.0) | 2.2 (2.1-2.3) |
|  |  | P < 0.001 | P < 0.001 |  | P < 0.001 | P < 0.001 |  | P < 0.001 | P < 0.001 |
| **LOS>7-day** | Reference | 2.2 (2.1-2.3) | 2.7 (2.6-2.9) | Reference | 2.8 (2.7-2.9) | 4.7 (4.6-4.8) | Reference | 2.3 (2.2-2.4) | 3.0 (2.9-3.2) |
|  |  | P < 0.001 | P < 0.001 |  | P < 0.001 | P < 0.001 |  | P < 0.001 | P < 0.001 |
| **LOS>10-day** | Reference | 2.4 (2.3-2.5) | 3.1 (3.0-3.3) | Reference | 3.0 (2.9-3.1) | 5.1 (4.9-5.3) | Reference | 2.5 (2.4-2.6) | 3.4 (3.2-3.6) |
|  |  | P < 0.001 | P < 0.001 |  | P < 0.001 | P < 0.001 |  | P < 0.001 | P < 0.001 |
| **LOS>14-day** | Reference | 2.6 (2.5-2.8) | 3.5 (3.3-3.7) | Reference | 3.3 (3.2-3.4) | 5.5 (5.3-5.7) | Reference | 2.8 (2.6-2.9) | 3.8 (3.6-4.0) |
|  |  | P < 0.001 | P < 0.001 |  | P < 0.001 | P < 0.001 |  | P < 0.001 | P < 0.001 |
| **LOS>21-day** | Reference | 3.0 (2.8-3.2) | 4.2 (3.9-4.5) | Reference | 3.6 (3.4-3.8) | 6.3 (6.0-6.6) | Reference | 3.1 (2.9-3.3) | 4.4 (4.1-4.8) |
|  |  | P < 0.001 | P < 0.001 |  | P < 0.001 | P < 0.001 |  | P < 0.001 | P < 0.001 |
| **LOS>30-day** | Reference | 3.2 (2.9-3.5) | 5.0 (4.5-5.5) | Reference | 3.7 (3.5-4.0) | 6.8 (6.5-7.2) | Reference | 3.2 (2.9-3.6) | 5.1 (4.6-5.7) |
|  |  | P < 0.001 | P < 0.001 |  | P < 0.001 | P < 0.001 |  | P < 0.001 | P < 0.001 |
| **LOS>45-day** | Reference | 3.5 (3.0-4.1) | 5.1 (4.4-6.0) | Reference | 3.8 (3.5-4.1) | 7.9 (6.4-9.6) | Reference | 3.5 (3.0-4.1) | 5.2 (4.6-6.3) |
|  |  | P < 0.001 | P < 0.001 |  | P < 0.001 | P < 0.001 |  | P < 0.001 | P < 0.001 |
| **LOS>60-day** | Reference | 3.8 (3.0-4.9) | 5.1 (3.9-6.5) | Reference | 4.3 (3.8-4.9) | 9.4 (6.5-11.4) | Reference | 3.8 (2.9-4.8) | 5.1 (3.9-6.6) |
|  |  | P < 0.001 | P < 0.001 |  | P < 0.001 | P < 0.001 |  | P < 0.001 | P < 0.001 |
| **LOS>90-day** | Reference | 4.6 (2.6-8.7) | 6.7 (3.7-12.8) | Reference | 5.2 (4.0-6.8) | 11.4 (7.3-13.2) | Reference | 5.5 (3.0-7.1) | 7.5 (4.2-9.8) |
|  |  | P < 0.001 | P < 0.001 |  | P < 0.001 | P < 0.001 |  | P < 0.001 | P < 0.001 |
| **3 day-mortality** | Reference | 1.3 (1.2-1.5) | 1.3 (1.0-1.3) | Reference | 1.6 (1.4-1.7) | 1.5 (1.3-1.7) | Reference | 1.5 (1.3-1.7) | 1.4 (1.2-1.6) |
|  |  | P < 0.001 | P < 0.001 |  | P < 0.001 | P < 0.001 |  | P < 0.001 | P < 0.001 |
| **7 day-mortality** | Reference | 1.5 (1.3-1.6) | 1.3 (1.1-1.4) | Reference | 1.9 (1.7-2.0) | 1.8 (1.6-1.9) | Reference | 1.6 (1.4-1.8) | 1.5 (1.3-1.7) |
|  |  | P < 0.001 | P < 0.001 |  | P < 0.001 | P < 0.001 |  | P < 0.001 | P < 0.001 |
| **10 day-mortality** | Reference | 1.5 (1.4-1.6) | 1.3 (1.2-1.5) | Reference | 2.0 (1.8-2.1) | 1.9 (1.8-2.1) | Reference | 1.7 (1.5-1.9) | 1.6 (1.4-1.8) |
|  |  | P < 0.001 | P < 0.001 |  | P < 0.001 | P < 0.001 |  | P < 0.001 | P < 0.001 |
| **14 day-mortality** | Reference | 1.6 (1.4-1.7) | 1.4 (1.3-1.5) | Reference | 2.1 (1.9-2.2) | 2.2 (1.9-2.3) | Reference | 1.7 (1.6-1.9) | 1.6 (1.4-1.8) |
|  |  | P < 0.001 | P < 0.001 |  | P < 0.001 | P < 0.001 |  | P < 0.001 | P < 0.001 |
| **30 day-mortality** | Reference | 1.6 (1.5-1.7) | 1.5 (1.4-1.6) | Reference | 2.2 (2.1-2.7) | 2.6 (2.4-2.7) | Reference | 1.8 (1.6-2.0) | 1.8 (1.6-2.0) |
|  |  | P < 0.001 | P < 0.001 |  | P < 0.001 | P < 0.001 |  | P < 0.001 | P < 0.001 |
| **60 day-mortality** | Reference | 1.6 (1.5-1.8) | 1.5 (1.4-1.7) | Reference | 2.4 (2.3-2.5) | 2.9 (2.7-3.1) | Reference | 1.9 (1.7-2.0) | 1.9 (1.7-2.1) |
|  |  | P < 0.001 | P < 0.001 |  | P < 0.001 | P < 0.001 |  | P < 0.001 | P < 0.001 |
| **90 day-mortality** | Reference | 1.7 (1.5-1.8) | 1.6 (1.4-1.7) | Reference | 2.4 (2.3-2.6) | 3.2 (2.8-3.8) | Reference | 1.9 (1.7-2.0) | 1.9 (1.7-2.1) |
|  |  | P < 0.001 | P < 0.001 |  | P < 0.001 | P < 0.001 |  | P < 0.001 | P < 0.001 |
| **6month-mortality** | Reference | 1.7 (1.5-1.8) | 1.6 (1.4-1.7) | Reference | 2.4 (2.3-2.6) | 3.5 (2.8-4.2) | Reference | 1.9 (1.7-2.0) | 1.9 (1.7-2.1) |
|  |  | P < 0.001 | P < 0.001 |  | P < 0.001 | P < 0.001 |  | P < 0.001 | P < 0.001 |

modified HFRS*: calculate modified HFRS by excluding the index admission into the HFRS calculation

S3-b Table. Results of logistic regression with interaction models for modified HFRS* and poor outcomes among patient with the probability of sepsis (based on SOS codes and NEWS≥7) For patients aged from 50-74 years

|  | **Group A: SOS code-present** | | | **Group B: NEWS≥7** | | | **Group C: SOS codes-present with NEWS≥7** | | |
| --- | --- | --- | --- | --- | --- | --- | --- | --- | --- |
| **outcomes** | **Odds Ratio (95% CI)** | | | **Odds Ratio (95% CI)** | | | **Odds Ratio (95% CI)** | | |
|  | **Interaction HFRS: sepsis-risk-positive (P- value)** | | | **Interaction HFRS: sepsis-risk-positive (P- value)** | | | **Interaction HFRS: sepsis-risk-positive (P- value)** | | |
|  | **Low frailty risk and SOS codes-absent** | **Intermediate frailty risk** | **High frailty risk** | **Low frailty risk and NEWS<7** | **Intermediate frailty risk** | **High frailty risk** | **Low frailty risk, SOS codes-absent and NEWS<7** | **Intermediate frailty risk** | **High frailty risk** |
| **LOS> 3-day** | Reference | 2.0 (1.8-2.0) | 2.2 (2.1-2.3) | Reference | 2.5 (2.4-2.6) | 3.8 (3.5-4.0) | Reference | 2.0 (1.9-2.1) | 2.4 (2.2-2.5) |
|  |  | P < 0.001 | P < 0.001 |  | P < 0.001 | P < 0.001 |  | P < 0.001 | P < 0.001 |
| **LOS>7-day** | Reference | 2.3 (2.2-2.4) | 3.0 (2.8-3.2) | Reference | 2.9 (2.8-3.0) | 4.9 (4.6-5.1) | Reference | 2.3 (2.2-2.5) | 3.1 (2.9-3.4) |
|  |  | P < 0.001 | P < 0.001 |  | P < 0.001 | P < 0.001 |  | P < 0.001 | P < 0.001 |
| **LOS>10-day** | Reference | 2.6 (2.4-2.7) | 3.6 (3.3-3.9) | Reference | 3.2 (3.1-3.4) | 5.6 (5.3-5.9) | Reference | 2.6 (2.4-2.7) | 3.7 (3.4-4.0) |
|  |  | P < 0.001 | P < 0.001 |  | P < 0.001 | P < 0.001 |  | P < 0.001 | P < 0.001 |
| **LOS>14-day** | Reference | 2.8 (2.6-3.0) | 4.0 (3.7-4.5) | Reference | 3.5 (3.4-3.7) | 6.3 (6.0-6.7) | Reference | 2.8 (2.6-3.0) | 4.2 (3.7-4.5) |
|  |  | P < 0.001 | P < 0.001 |  | P < 0.001 | P < 0.001 |  | P < 0.001 | P < 0.001 |
| **LOS>21-day** | Reference | 3.3 (2.9-3.6) | 4.8 (4.2-5.5) | Reference | 4.0 (3.7-4.2) | 7.4 (6.9-7.9) | Reference | 3.2 (2.9-3.6) | 4.8 (4.3-5.5) |
|  |  | P < 0.001 | P < 0.001 |  | P < 0.001 | P < 0.001 |  | P < 0.001 | P < 0.001 |
| **LOS>30-day** | Reference | 3.6 (3.1-4.1) | 5.5 (4.6-6.6) | Reference | 4.3 (4.0-4.7) | 8.0 (7.4-8.8) | Reference | 3.6 (3.1-4.1) | 5.6 (4.6-6.6) |
|  |  | P < 0.001 | P < 0.001 |  | P < 0.001 | P < 0.001 |  | P < 0.001 | P < 0.001 |
| **LOS>45-day** | Reference | 4.4 (3.6-5.5) | 6.0 (5.5-8.2) | Reference | 5.0 (4.5-5.7) | 9.7 (8.6-11.0) | Reference | 4.3 (3.4-5.3) | 7.0 (5.4-9.1) |
|  |  | P < 0.001 | P < 0.001 |  | P < 0.001 | P < 0.001 |  | P < 0.001 | P < 0.001 |
| **LOS>60-day** | Reference | 4.6 (3.5-6.2) | 6.3 (4.3-8.5) | Reference | 5.7 (4.8-6.7) | 10.4 (8.8-12.3) | Reference | 4.4 (3.3-5.8) | 7.1 (5.4-8.4) |
|  |  | P < 0.001 | P < 0.001 |  | P < 0.001 | P < 0.001 |  | P < 0.001 | P < 0.001 |
| **LOS>90-day** | Reference | 7.2 (5.5-9.2) | 10.5 (6.5-15.2) | Reference | 7.9 (5.9-10.6) | 15.3 (9.2-18.5) | Reference | 8.8 (4.8-11.9) | 10.6 (5.1-16.3) |
|  |  | P < 0.001 | P < 0.001 |  | P < 0.001 | P < 0.001 |  | P < 0.001 | P < 0.001 |
| **3 day-mortality** | Reference | 1.9 (1.4-2.0) | 1.6 (1.0-1.8) | Reference | 2.1 (1.7-2.4) | 2.3 (1.8-2.9) | Reference | 1.8 (1.4-2.3) | 1.6 (0.7-1.7) |
|  |  | P < 0.001 | P < 0.001 |  | P < 0.001 | P < 0.001 |  | P < 0.001 | P < 0.001 |
| **7 day-mortality** | Reference | 2.0 (1.7-2.3) | 1.7 (1.2-2.0) | Reference | 2.2 (1.9-2.5) | 2.6 (2.2-3.0) | Reference | 2.1 (1.7-2.5) | 1.7 (1.1-2.0) |
|  |  | P < 0.001 | P < 0.001 |  | P < 0.001 | P < 0.001 |  | P < 0.001 | P < 0.001 |
| **10 day-mortality** | Reference | 2.1 (1.8-2.4) | 1.8 (1.4-2.1) | Reference | 2.3 (2.1-2.6) | 2.7 (2.4-3.1) | Reference | 2.1 (1.8-2.5) | 1.7 (1.3-2.2) |
|  |  | P < 0.001 | P < 0.001 |  | P < 0.001 | P < 0.001 |  | P < 0.001 | P < 0.001 |
| **14 day-mortality** | Reference | 2.1 (1.9-2.4) | 1.9 (1.5-2.3) | Reference | 2.6 (2.3-2.8) | 2.8 (2.4-3.1) | Reference | 2.2 (1.9-2.6) | 1.8 (1.4-2.3) |
|  |  | P < 0.001 | P < 0.001 |  | P < 0.001 | P < 0.001 |  | P < 0.001 | P < 0.001 |
| **30 day-mortality** | Reference | 2.2 (1.9-2.4) | 2.0 (1.6-2.3) | Reference | 2.8 (2.5-3.0) | 3.9 (3.1-4.2) | Reference | 2.3 (1.9-2.6) | 2.0 (1.6-2.5) |
|  |  | P < 0.001 | P < 0.001 |  | P < 0.001 | P < 0.001 |  | P < 0.001 | P < 0.001 |
| **60 day-mortality** | Reference | 2.2 (1.9-2.4) | 2.0 (1.6-2.3) | Reference | 2.9 (2.6-3.2) | 3.9 (3.1-4.2) | Reference | 2.3 (1.9-2.6) | 2.1 (1.6-2.6) |
|  |  | P < 0.001 | P < 0.001 |  | P < 0.001 | P < 0.001 |  | P < 0.001 | P < 0.001 |
| **90 day-mortality** | Reference | 2.2 (1.9-2.4) | 2.0 (1.7-2.3) | Reference | 3.0 (2.7-3.2) | 4.0 (3.5-4.4) | Reference | 2.3 (1.9-2.6) | 2.1 (1.6-2.6) |
|  |  | P < 0.001 | P < 0.001 |  | P < 0.001 | P < 0.001 |  | P < 0.001 | P < 0.001 |
| **6month-mortality** | Reference | 2.2 (1.9-2.4) | 2.1 (1.7-2.3) | Reference | 3.0 (2.7-3.2) | 4.0 (3.5-4.4) | Reference | 2.3 (1.9-2.6) | 2.1 (1.6-2.6) |
|  |  | P < 0.001 | P < 0.001 |  | P < 0.001 | P < 0.001 |  | P < 0.001 | P < 0.001 |

modified HFRS*: calculate modified HFRS by excluding the index admission into the HFRS calculation

S3-c Table. Results of logistic regression with interaction models for modified HFRS* and poor outcomes among patient with the probability of sepsis (based on SOS codes and NEWS≥7) For patients aged ≥75

|  | **Group A: SOS code-present** | | | **Group B: NEWS≥7** | | | **Group C: SOS codes-present with NEWS≥7** | | |
| --- | --- | --- | --- | --- | --- | --- | --- | --- | --- |
| **outcomes** | **Odds Ratio (95% CI)** | | | **Odds Ratio (95% CI)** | | | **Odds Ratio (95% CI)** | | |
|  | **Interaction HFRS: sepsis-risk-positive (P- value)** | | | **Interaction HFRS: sepsis-risk-positive (P- value)** | | | **Interaction HFRS: sepsis-risk-positive (P- value)** | | |
|  | **Low frailty risk and SOS codes-absent** | **Intermediate frailty risk** | **High frailty risk** | **Low frailty risk and NEWS<7** | **Intermediate frailty risk** | **High frailty risk** | **Low frailty risk, SOS codes-absent and NEWS<7** | **Intermediate frailty risk** | **High frailty risk** |
| **LOS> 3-day** | Reference | 2.1 (1.6-2.2) | 2.3 (1.8-2.4) | Reference | 2.3 (2.2-2.4) | 3.5 (3.4-3.6) | Reference | 2.0 (1.9-2.1) | 2.1 (2.0-2.2) |
|  |  | P < 0.001 | P < 0.001 |  | P < 0.001 | P < 0.001 |  | P < 0.001 | P < 0.001 |
| **LOS>7-day** | Reference | 2.1 (2.0-2.2) | 2.5 (2.4-2.6) | Reference | 2.7 (2.6-2.8) | 4.3 (4.2-4.4) | Reference | 2.3 (2.2-2.4) | 2.8 (2.6-2.9) |
|  |  | P < 0.001 | P < 0.001 |  | P < 0.001 | P < 0.001 |  | P < 0.001 | P < 0.001 |
| **LOS>10-day** | Reference | 2.3 (2.2-2.4) | 2.8 (2.7-2.9) | Reference | 2.9 (2.7-3.0) | 4.6 (4.4-4.7) | Reference | 2.4 (2.3-2.5) | 3.1 (2.9-3.2) |
|  |  | P < 0.001 | P < 0.001 |  | P < 0.001 | P < 0.001 |  | P < 0.001 | P < 0.001 |
| **LOS>14-day** | Reference | 2.5 (2.4-2.7) | 3.2 (3.0-3.4) | Reference | 3.1 (2.9-3.2) | 4.9 (4.7-5.1) | Reference | 2.6 (2.5-2.7) | 3.4 (3.2-3.6) |
|  |  | P < 0.001 | P < 0.001 |  | P < 0.001 | P < 0.001 |  | P < 0.001 | P < 0.001 |
| **LOS>21-day** | Reference | 2.7 (2.5-2.9) | 3.7 (3.4-4.0) | Reference | 3.3 (3.1-3.5) | 5.5 (5.2-5.7) | Reference | 2.8 (2.6-3.1) | 3.9 (3.6-4.3) |
|  |  | P < 0.001 | P < 0.001 |  | P < 0.001 | P < 0.001 |  | P < 0.001 | P < 0.001 |
| **LOS>30-day** | Reference | 2.9 (2.6-3.3) | 4.4 (3.9-4.9) | Reference | 3.5 (3.2-3.7) | 6.1 (5.7-6.5) | Reference | 3.0 (2.6-3.3) | 4.5 (4.0-5.1) |
|  |  | P < 0.001 | P < 0.001 |  | P < 0.001 | P < 0.001 |  | P < 0.001 | P < 0.001 |
| **LOS>45-day** | Reference | 3.0 (2.5-3.5) | 4.4 (3.9-4.9) | Reference | 3.6 (3.2-3.8) | 6.5 (5.8-6.8) | Reference | 3.3 (2.6-3.6) | 4.7 (4.2-5.2) |
|  |  | P < 0.001 | P < 0.001 |  | P < 0.001 | P < 0.001 |  | P < 0.001 | P < 0.001 |
| **LOS>60-day** | Reference | 3.4 (2.6-4.5) | 4.7 (3.4-6.2) | Reference | 4.1 (3.5-4.8) | 6.8 (5.8-7.9) | Reference | 3.5 (2.6-4.2) | 5.3 (4.0-6.3) |
|  |  | P < 0.001 | P < 0.001 |  | P < 0.001 | P < 0.001 |  | P < 0.001 | P < 0.001 |
| **LOS>90-day** | Reference | 3.4 (2.6-4.5) | 4.8 (2.5-7.8) | Reference | 5.7 (4.0-8.4) | 13.8 (7.3-16.8) | Reference | 3.8 (2.1-6.3) | 6.0 (3.3-10.3) |
|  |  | P < 0.001 | P < 0.001 |  | P < 0.001 | P < 0.001 |  | P < 0.001 | P < 0.001 |
| **3 day-mortality** | Reference | 1.5 (1.3-1.7) | 1.4 (1.3-1.5) | Reference | 1.4 (1.2-1.5) | 1.6 (1.5-1.7) | Reference | 1.3 (1.1-1.4) | 1.3 (1.2-1.5) |
|  |  | P < 0.001 | P < 0.001 |  | P < 0.001 | P < 0.001 |  | P < 0.001 | P < 0.001 |
| **7 day-mortality** | Reference | 1.8 (1.6-2.0) | 1.5 (1.4-1.6) | Reference | 1.7 (1.5-1.8) | 1.8 (1.6-1.9) | Reference | 1.4 (1.2-1.6) | 1.3 (1.2-1.5) |
|  |  | P < 0.001 | P < 0.001 |  | P < 0.001 | P < 0.001 |  | P < 0.001 | P < 0.001 |
| **10 day-mortality** | Reference | 2.0 (1.8-2.3) | 1.5 (1.4-1.6) | Reference | 1.8 (1.6-1.9) | 2.1 (2.0-2.3) | Reference | 1.5 (1.3-1.6) | 1.3 (1.2-1.5) |
|  |  | P < 0.001 | P < 0.001 |  | P < 0.001 | P < 0.001 |  | P < 0.001 | P < 0.001 |
| **14 day-mortality** | Reference | 2.3 (2.1-2.5) | 1.5 (1.4-1.6) | Reference | 1.8 (1.6-1.9) | 2.3 (2.1-2.4) | Reference | 1.5 (1.3-1.6) | 1.4 (1.2-1.6) |
|  |  | P < 0.001 | P < 0.001 |  | P < 0.001 | P < 0.001 |  | P < 0.001 | P < 0.001 |
| **30 day-mortality** | Reference | 2.6 (2.3-2.8) | 1.7 (1.5-1.8) | Reference | 2.0 (1.8-2.1) | 2.5 (2.3-2.6) | Reference | 1.6 (1.4-1.8) | 1.6 (1.4-1.7) |
|  |  | P < 0.001 | P < 0.001 |  | P < 0.001 | P < 0.001 |  | P < 0.001 | P < 0.001 |
| **60 day-mortality** | Reference | 2.7 (2.5-2.9) | 1.7 (1.5-1.8) | Reference | 2.1 (2.0-2.3) | 2.9 (2.6-2.9) | Reference | 1.6 (1.4-1.8) | 1.6 (1.4-1.7) |
|  |  | P < 0.001 | P < 0.001 |  | P < 0.001 | P < 0.001 |  | P < 0.001 | P < 0.001 |
| **90 day-mortality** | Reference | 2.8 (2.6-3.0) | 1.9 (1.3-2.1) | Reference | 2.1 (2.0-2.3) | 3.3 (2.9-3.2) | Reference | 1.7 (1.5-1.8) | 1.7 (1.5-1.9) |
|  |  | P < 0.001 | P < 0.001 |  | P < 0.001 | P < 0.001 |  | P < 0.001 | P < 0.001 |
| **6month-mortality** | Reference | 2.9 (2.6-3.0) | 1.9 (1.3-2.1) | Reference | 2.1 (2.0-2.3) | 3.6 (3.3-3.9) | Reference | 1.7 (1.5-1.8) | 1.7 (1.5-1.9) |
|  |  | P < 0.001 | P < 0.001 |  | P < 0.001 | P < 0.001 |  | P < 0.001 | P < 0.001 |

modified HFRS*: calculate modified HFRS by excluding the index admission into the HFRS calculation
