# Supplementary figures and images for: Association between hospital frailty risk score, risk of sepsis and adverse outcomes across all adult ages
Source: PLoS One. 2026 Feb 13;21(2):e0342790. doi: 10.1371/journal.pone.0342790 (PMC12904455; doi:10.1371/journal.pone.0342790)

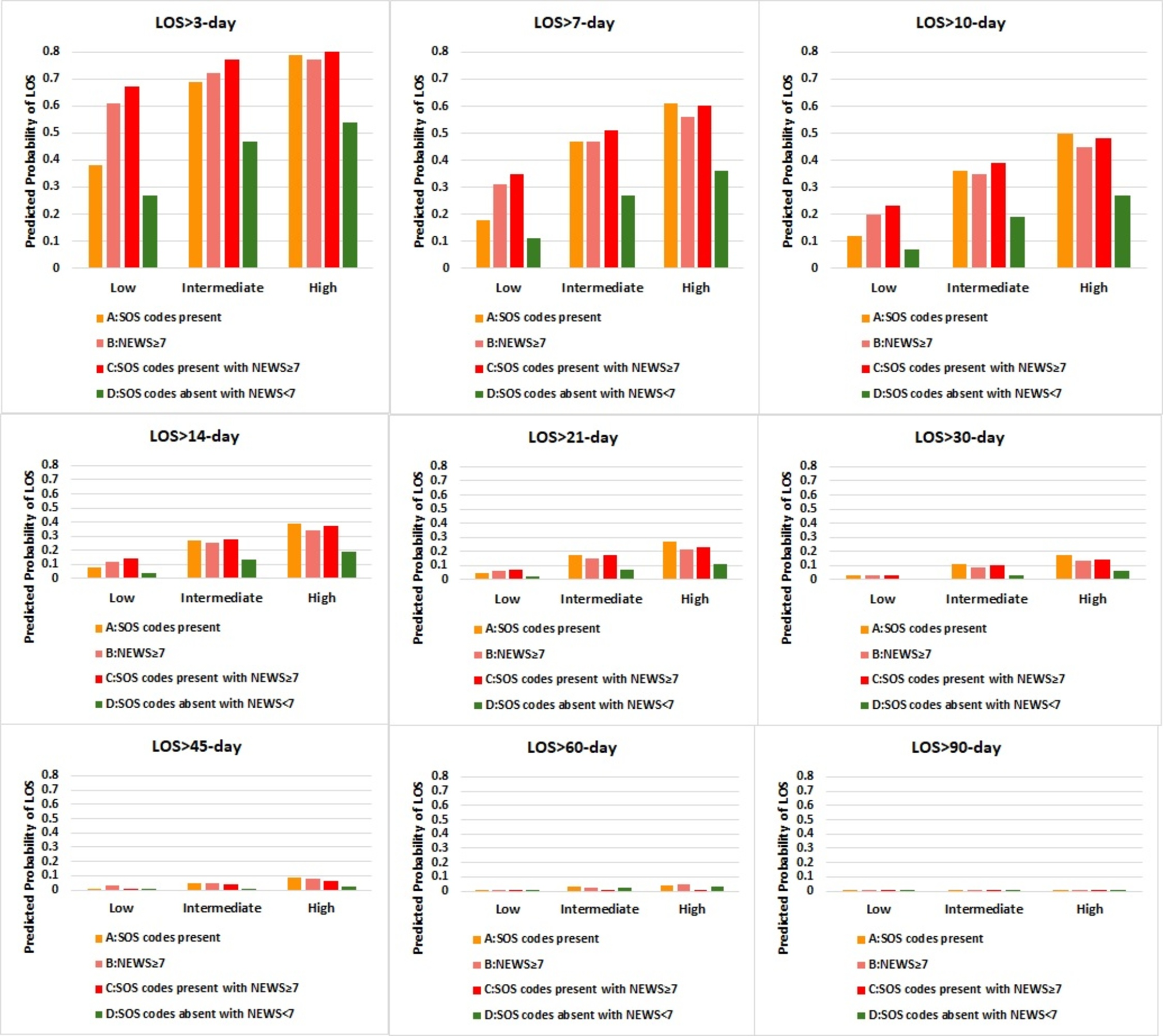

Supplement: S1 Fig — (TIF) [file pone.0342790.s005.tif]

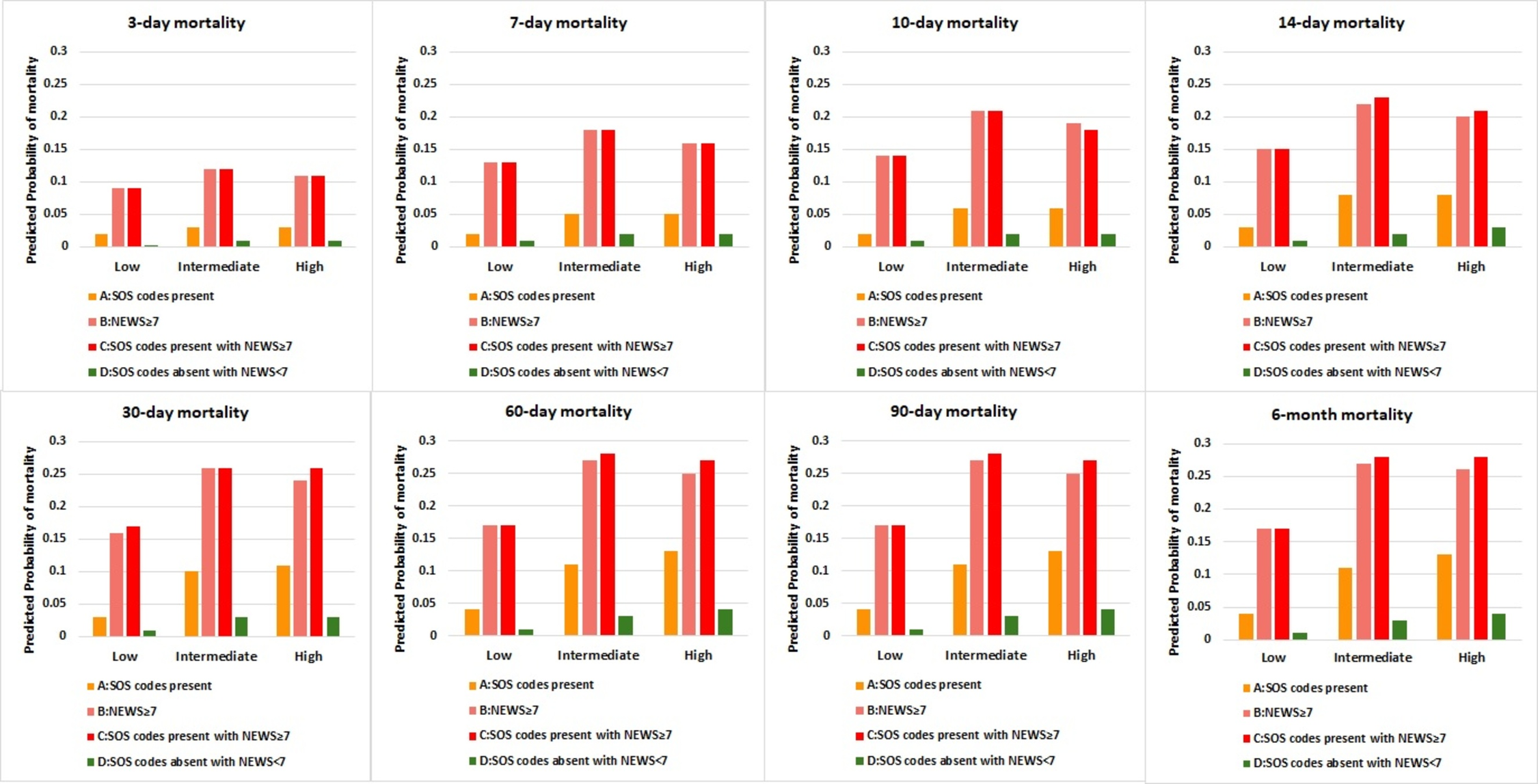

Supplement: S2 Fig — (TIF) [file pone.0342790.s006.tif]

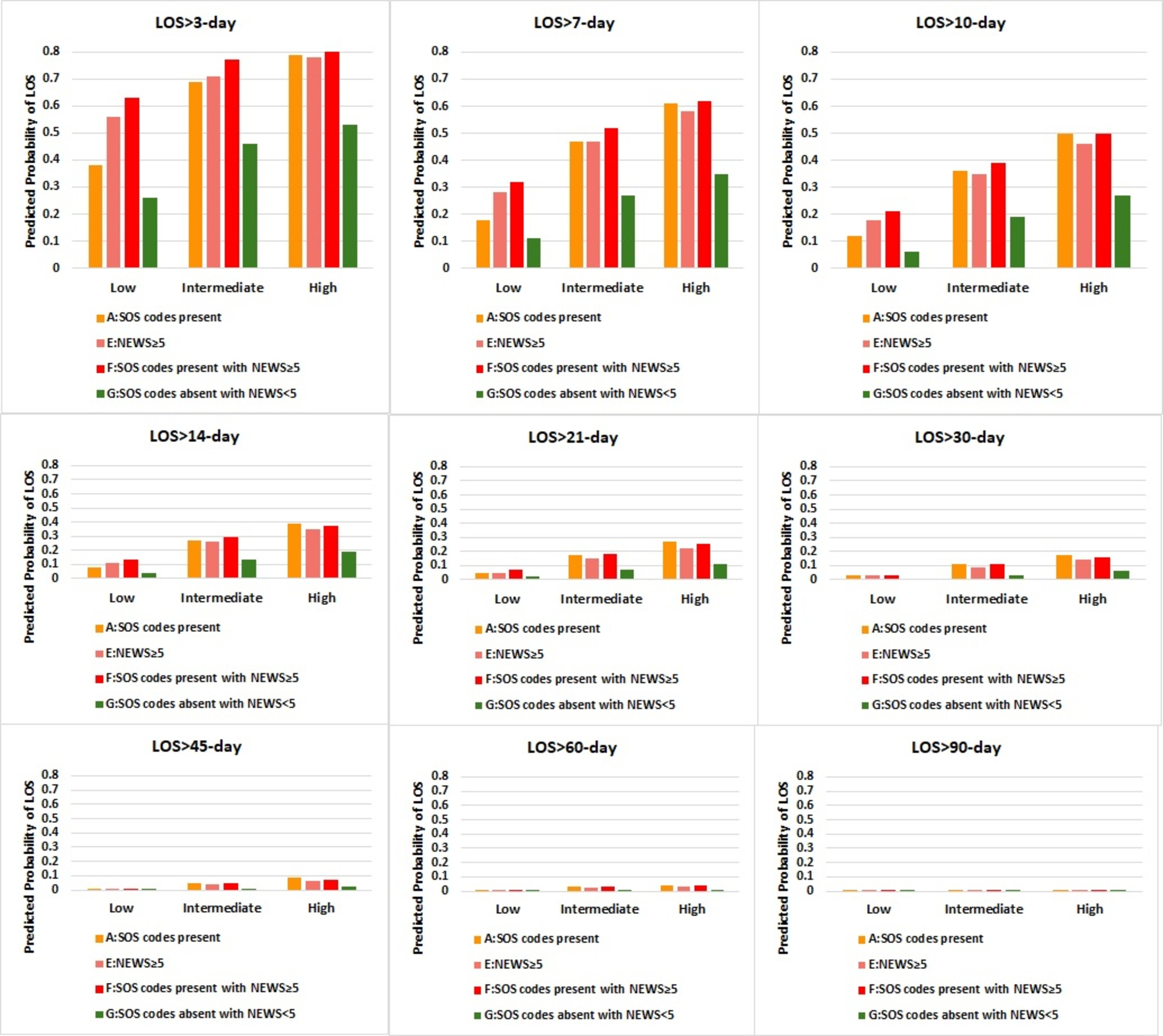

Supplement: S3 Fig — (TIF) [file pone.0342790.s007.tif]

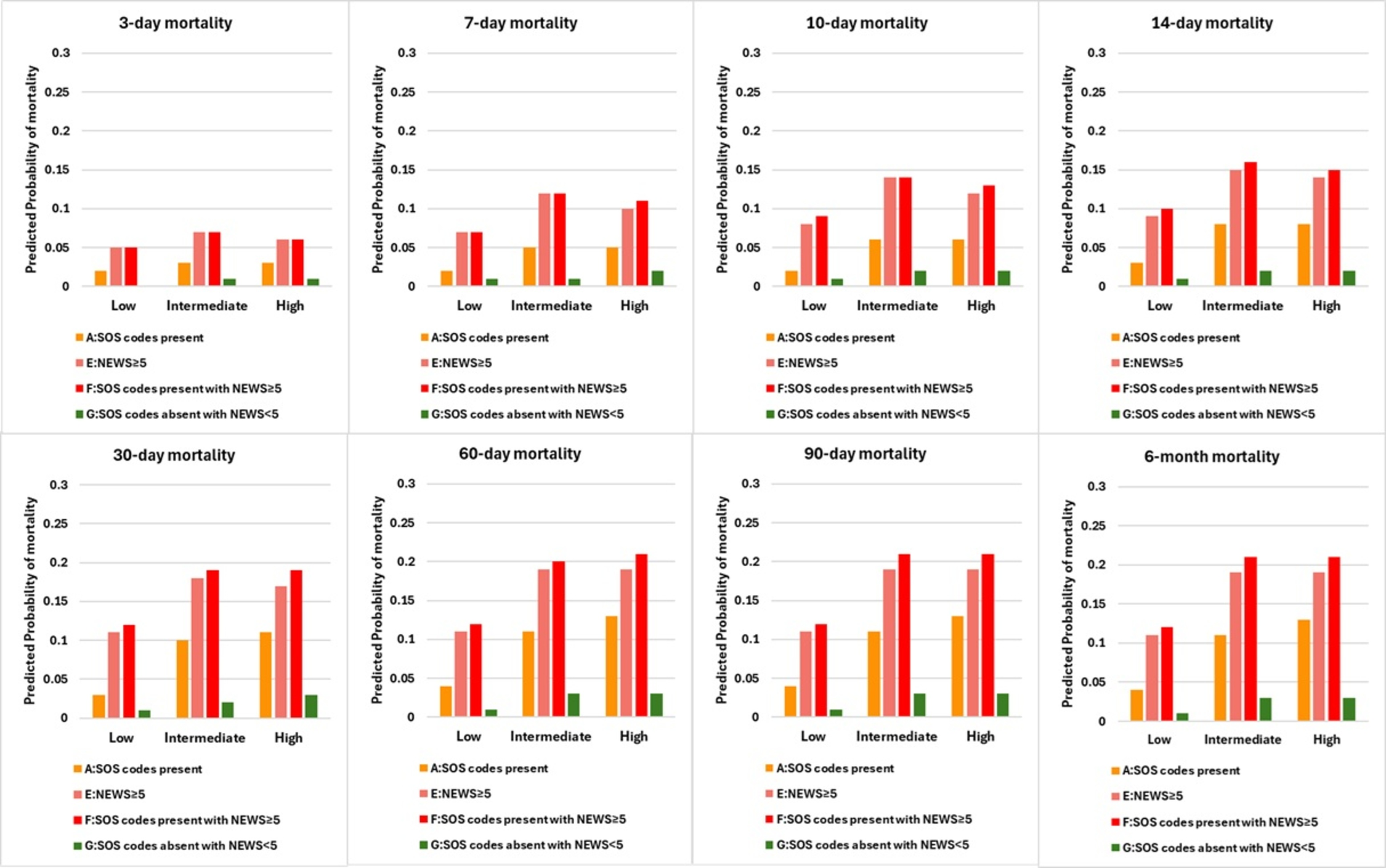

Supplement: S4 Fig — (TIF) [file pone.0342790.s008.tif]
